# Homology-directed repair in rodent zygotes using Cas9 and TALEN engineered proteins

**DOI:** 10.1038/srep14410

**Published:** 2015-10-07

**Authors:** Séverine Ménoret, Anne De Cian, Laurent Tesson, Séverine Remy, Claire Usal, Jean-Baptiste Boulé, Charlotte Boix, Sandra Fontanière, Alison Crénéguy, Tuan H. Nguyen, Lucas Brusselle, Reynald Thinard, Dominique Gauguier, Jean-Paul Concordet, Yacine Cherifi, Alexandre Fraichard, Carine Giovannangeli, Ignacio Anegon

**Affiliations:** 1 INSERM UMR 1064-ITUN; CHU de Nantes, Nantes F44093, France; 2 Platform Rat Transgenesis Immunophenomic, SFR François Bonamy, CNRS UMS3556 Nantes, F44093, France; 3 INSERM U1154, CNRS UMR7196, Structure and Instability of Genomes, Sorbonne Universités, Museum National d’Histoire Naturelle; CP26 57 rue Cuvier, F75005 Paris, France; 4 genOway, Lyon F69007, France; 5 Sorbonne Universities, University Pierre & Marie Curie, University Paris Descartes, Sorbonne Paris Cité, INSERM UMR_S 1138, Cordeliers Research Centre, 75006 Paris, France.; 6 Institute of Cardiometabolism and Nutrition, University Pierre & Marie Curie, Boulevard de l’Hopital, 75013 Paris, France

## Abstract

The generation of genetically-modified organisms has been revolutionized by the development of new genome editing technologies based on the use of gene-specific nucleases, such as meganucleases, ZFNs, TALENs and CRISPRs-Cas9 systems. The most rapid and cost-effective way to generate genetically-modified animals is by microinjection of the nucleic acids encoding gene-specific nucleases into zygotes. However, the efficiency of the procedure can still be improved. In this work we aim to increase the efficiency of CRISPRs-Cas9 and TALENs homology-directed repair by using TALENs and Cas9 proteins, instead of mRNA, microinjected into rat and mouse zygotes along with long or short donor DNAs. We observed that Cas9 protein was more efficient at homology-directed repair than mRNA, while TALEN protein was less efficient than mRNA at inducing homology-directed repair. Our results indicate that the use of Cas9 protein could represent a simple and practical methodological alternative to Cas9 mRNA in the generation of genetically-modified rats and mice as well as probably some other mammals.

Engineered nucleases, such as zinc-finger nucleases (ZFNs), transcription activator-like effector nucleases (TALENs) and clustered regularly interspaced short palindromic repeats (CRISPR)/CRISPR associated protein 9 (Cas9) are key tools for the introduction of gene-inactivating mutations by non-homologous end joining (NHEJ) mechanisms, as well as precise modifications such as base mutation, exon exchange or insertion of expression cassettes by homology-directed recombination (HDR)^1^,^2^. Engineered nucleases injected into zygotes as nucleic acids have been particularly useful for the generation of genetically modified animals in several species in which it was previously impossible or very difficult due to the absence of robust ES cells^2^,^3^. This is particularly the case in the rat, an important animal model^4^,^5^ that has been successfully genetically modified to create new disease models^4^,^6^,^7^,^8^ as well as biotechnological applications^9^. The CRISPR/Cas9 system using Cas9 mRNA has been used to generate genetically-engineered rats^10^,^11^,^12^ and mice^13^,^14^. However, the HDR frequencies are low and increasing them would accelerate and facilitate the generation of genetically modified animals useful in the analysis of genes, creation of disease models and production of recombinant proteins^1^,^2^,^5^.

We reasoned that if the engineered nucleases could be delivered as proteins it would increase HDR efficiency since the transcription and/or translation steps would be avoided and degradation of the gene targeting construct limited, which would increase its availability. To the best of our knowledge, only one publication describes the use of Cas9 protein to obtain integration of short sequences, using ssODNs in zebrafish^15^, and two other publications gene invalidation by NHEJ, in zebrafish and mice^16^ and *C. elegans*^17^. Every other genetically engineered animal model, including rats and mice, using any type of nuclease, including meganucleases, ZFNs and TALENs, has been generated using DNA or mRNA^2^,^3^, TALEN proteins, in particular, have never been used in the generation of transgenic animals. In this context, we recently described HDR in the rat *Rosa26* locus using TALEN mRNA and a large GFP expression cassette^1^. However, improving the targeted integration of long transgenes by HDR remains a challenge and the benefit of using nucleases directly as proteins has not been tested. In the present work, we generated and purified Cas9 protein and two TALEN monomers targeting the rat *Rosa26* locus (for the TALEN proteins, they correspond to those expressed from mRNA used previously to obtain HDR). We microinjected into rat zygotes these engineered protein nucleases along with a long donor DNA fragment encoding for GFP, previously used to obtain HDR using TALEN encoded by mRNA^1^. Using Cas9 protein we observed higher efficiency in NHEJ and HDR compared to Cas9 mRNA. In contrast, TALEN proteins were less efficient than TALEN mRNA. We also targeted the mouse *Rosa26* locus using Cas9 protein and mRNA and a long donor DNA fragment and also observed a higher efficacy of Cas9 protein at generating HDR. Cas9 protein was also found to be more efficient at generating HDR compared to Cas9 mRNA in two more rat loci targeted, *Foxp3 and Anks3*, using, respectively, a long donor DNA fragment and ssODNs.

In conclusion, our results suggest that in rats and mice the use of Cas9 protein may be more efficient than mRNA at inducing HDR using both large and short DNA donor fragments. The use of Cas9 protein may widen the application of genome-editing techniques in different species.

## Results

### Generation of TALEN and Cas9 proteins and validation of their DNA cleavage in rat cells

**Figure 1:**
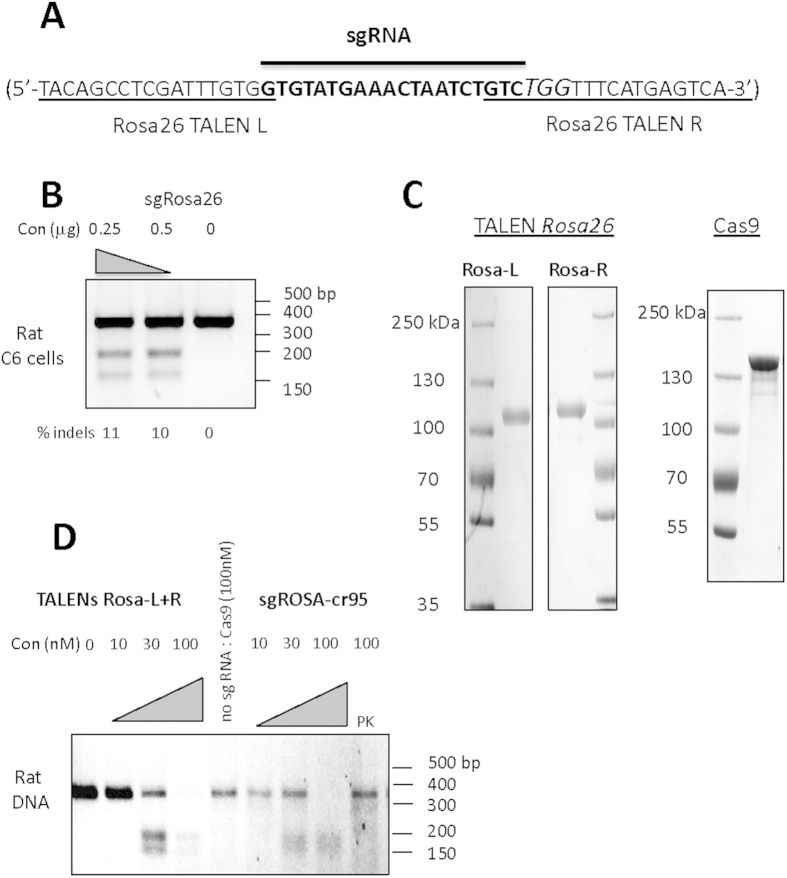
Production, *in vitro* and cellular activities of TALEN and Cas9 proteins. (**A**) Targeted sequences. The rat *Rosa26* target sequence with the TALEN binding sites underlined, the PAM in italics and larger size font, and the sgRNA binding sites in bold. (**B**) Cas9:sgRNA mutagenic activity in rat cells. Cas9 mRNAs (0.25 or 0.5 μg of each) and sgRNA were transfected in rat C6 cells and the mutation rate evaluated by the T7 endonuclease I assay. The fraction of amplified DNA (351 bp) cleaved by the T7 endonuclease (165 and 186 bp) due to indels is indicated as % indels. (**C**) Production of recombinant TALEN and Cas9. Two μg of each purified protein were analyzed on a 4–8% polyacrylamide SDS-PAGE gel and stained with Instantblue. (**D**) *In vitro* activity of recombinant TALEN proteins and sgRNA:Cas9 complexes on rat DNA. The rat *Rosa26* locus was PCR amplified (351 bp) and 10 nM of DNA was digested 1 hour at 37 °C in the presence of TALEN ROSA-L and ROSA-R proteins (10, 30 and 100 nM) or of a (1:2) sgRNA:Cas9 complex (10, 30, 100 nM) and run on agarose gels to analyze specific cleavage fragments (165 and 186 bp). Samples were treated with RNase and proteinase K (PK) before loading on an agarose gel. Controls of PK pre-treatment of sgRNA:Cas9 complex or Cas9 without sgRNA are shown for the specific activity of Cas9 protein in complex with the sgRNA.

The rat *Rosa26* loci were targeted in intron 1. TALEN proteins targeted sequences that had already been mutated using mRNA and were generated *in vitro* from mRNA (Fig. 1A)^1^. Cas9 protein was generated *in vitro* and single guided RNA (sgRNA) targeting sequences in between those recognized by the TALENs at the rat *Rosa26* locus were designed (Fig. 1A) and confirmed as functional by transfecting rat cells (Fig. 1B). The frequencies of indel generation (Fig. 1B) were in the range of efficiency obtained in other loci in our hands with this technology and were among the highest after testing several sgRNAs for this portion of the rat *Rosa26* locus (data not shown). TALENs and Cas9 proteins (expected size of 160 kDa pour Cas9 and 110 kDa for TALENs) were purified and showed to be largely clear of contaminants (Fig. 1C). With both TALEN and Cas9 purified proteins we observed efficient cleavage of rat *Rosa26* sequences *in vitro* (Fig. 1D).

### Microinjection into rat zygotes of TALEN and Cas9 proteins or mRNA targeting the *Rosa26* locus along with a long donor DNA fragment to obtain HDR

**Figure 2:**
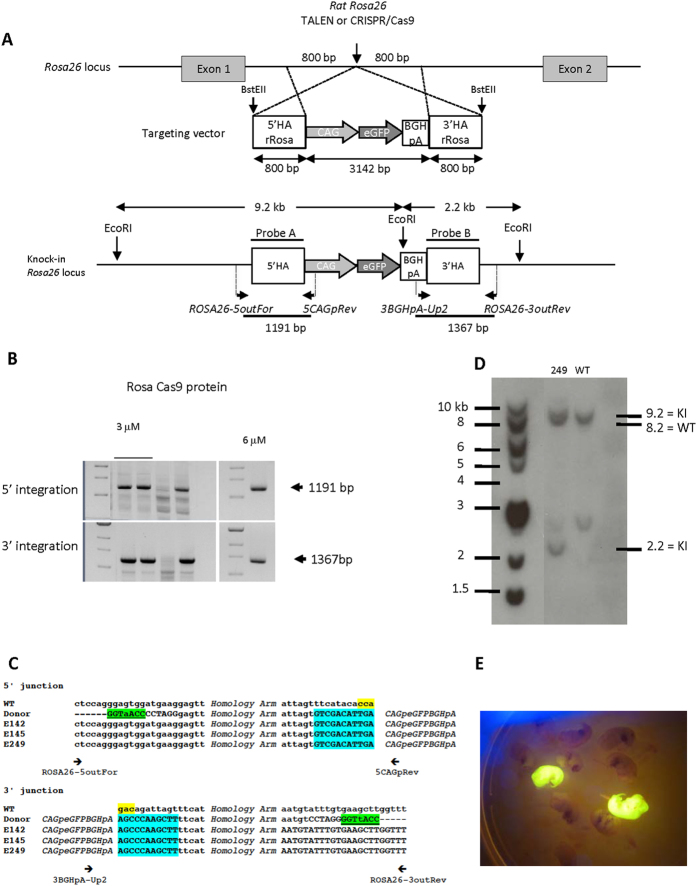
Targeted integration of a GFP cassette into the rat Rosa26 locus. (**A, upper**) Schematic representation of the rat *Rosa26* locus. TALEN and CRISPR/Cas9 action (vertical arrow), the targeting vector with expression cassette (3142 bp) and the 5′ and 3′ homology arms (HA) (800 bp each) are indicated. BstEII restriction sites are indicated. **(A**, lower) Schematic representation of the GFP cassette integration. Genomic DNAs were PCR amplified with primers situated for the 5′ side upstream of the 5′HA (ROSA26-5outFor) and in the CAG promoter (5CAGpRev), and for the 3′ side in the BGHpA (3BGHpA-Up2) and downstream the 3′ HA (ROSA26-3outRev). For Southern blot analysis, genomic DNA was digested with EcoRI and was probed with a sequence encompassing both homology arms. Two bands at 9.2 kb and 2.2 kb are predicted for a correct HDR in the *Rosa26* locus. (**B**) Flanking PCR analysis. The 3 GFP+ founders (142, 145 and 249) showed expected bands of 1191 bp using the first pair of primers (ROSA26-5outFor +5CAGpRev) and of 1367 bp using the second pair of primers (3BGHpA-Up2 + ROSA26-3outRev). A GFP negative E15 embryo (animal 240) is shown as negative control. The microinjection conditions in terms of protein are above each animal. (**C**) Analysis of flanking sequences. 5′ and 3′ junctions between wild-type genomic DNA and donor DNA sequences of the 3 GFP+ founders (142, 145, 249). In donor DNA, the presence of the BstEII site is indicated (underlined and colored in green). The 5′ and 3′ ends of the expression cassette are colored in blue. (**D**) Southern blot analysis. EcoRI digested DNA was analyzed with an homology arms probe (5′HA + 3′HA). One *Rosa26* GFP + rat was analyzed (animal 249) and showed 9.2 kb and 2.2 kb bands expected for a HDR with a single copy of the transgene. Both the HDR and a WT animal show a specific 8.2 kb (corresponding to a non-mutated allele in animal 249) and an additional non-specific band at 2.5 kb. (**E**) Two of the E15 *Rosa26* HDR rat fetuses (animals 142 and 145) show GFP expression under UV light while the others are negative.

We then microinjected the TALEN or Cas9 proteins along with a large donor DNA fragment comprising a GFP cassette and homologous sequences previously used to obtain HDR with TALENs as mRNA^1^ (Fig. 2A). Microinjection of rat zygotes was into the pronuclei and the cytoplasm using a mixture of either a Cas9/sgRNA protein complex (at equimolar concentration for both of 0.3, 3 or 6 μM), Cas9 mRNA/sgRNA or TALEN proteins (at 4 or 10 μM for both monomers) along with a long donor DNA fragment (4.7 Kb, 2 ng/μl). The donor DNA contained homology arms of 0.8 Kb and a CAG-GFP expression cassette previously shown to induce HDR in the rat *Rosa26* locus when used with TALENs mRNA^1^. The donor DNA was linearized before injection, since, as we have shown previously^1^, only linear, and not circular DNA, results in HDR. The use of this linear DNA resulted in the generation of transgenic rats by random integration but the circular form also generated transgenic rats at only slightly lower frequencies^1^. Day 15 fetuses were analyzed under UV light to detect GFP expression, and transgene integration was also confirmed by PCR amplification of the GFP coding sequence. HDR was determined by Southern blotting as well as by PCR using primers encompassing predicted integration sites followed by amplicons sequencing. NHEJ was analyzed by PCR of the targeted sequence followed by a T7 endonuclease I assay and sequencing of the amplicons.

Cas9 protein (complexed *in vitro* with sgRNA) injected at a concentration of 0.3 and 3 μM did not result in embryo toxicity but 6 μM resulted in a drastic reduction in the percentage of viable day 15 fetuses (Table 1). Cas9 protein at 0.3 μM resulted in neither HDR nor NHEJ mutations. Cas9 protein at 3 μM resulted in HDR in 1.7% and NHEJ in 11.1% of transferred eggs (*i.e.* 3.5% HDR and 22.8% NHEJ of day 15 embryos). At 6 μM, in spite of the marked toxicity, we observed some HDR (0.7%) and NHEJ (2.1%) (Table 1). Among fetuses microinjected at a concentration of 3 μM, two were confirmed as true HDR by positive junction PCRs (Fig. 2B) showing genomic sequences outside the homology arms (Fig. 2C) as well as bands of the expected size in Southern blots (Fig. 2D). Both fetuses positive for HDR showed GFP expression whereas the other fetuses were negative (Fig. 2E). In contrast, Cas9 mRNA and sgRNA failed to generate HDR and resulted in NHEJ in 2.35% of transferred eggs (or 7.55% of day 15 embryos) (Table 1). At 0.3 μM, where the Cas9 protein induced neither HDR nor NHEJ, several fetuses expressed GFP, but they contained randomly inserted DNA (Table 1).

Microinjection of TALEN proteins at a concentration of 8 and 20 μM TALENs generated NHEJ only in the higher concentration group, and there in 2.6% of microinjected embryos, and failed to generate HDR (Table 1). Precipitation of purified TALENs prevented microinjection at higher concentrations. Since TALEN mRNA (coding for the same TALENs) resulted in HDR in 2.2% and NHEJ in 3.3% of transferred eggs^1^, TALENs proteins in concentrations compatible with microinjection were less efficient than mRNA for both NHEJ and HDR.

NHEJ analysis of mutated founders showed an increased rate of mosaicism using Cas9 protein, since 42.1% of the fetuses were mosaics compared with 25% using Cas9 mRNA. It also tended to be higher using TALEN protein (33%) (data not shown) compared with TALEN mRNA (21.7%), as described in a previous work^1^.

These results show that for the rat *Rosa26* locus Cas9 protein but not TALEN proteins were more efficacious to obtaining HDR than the respective mRNA and this was associated to a higher rate of NHEJ.

### Microinjection into mouse zygotes of TALEN and Cas9 proteins or mRNA targeting the *Rosa26* locus along with a long donor DNA fragment to obtain HDR

**Figure 3:**
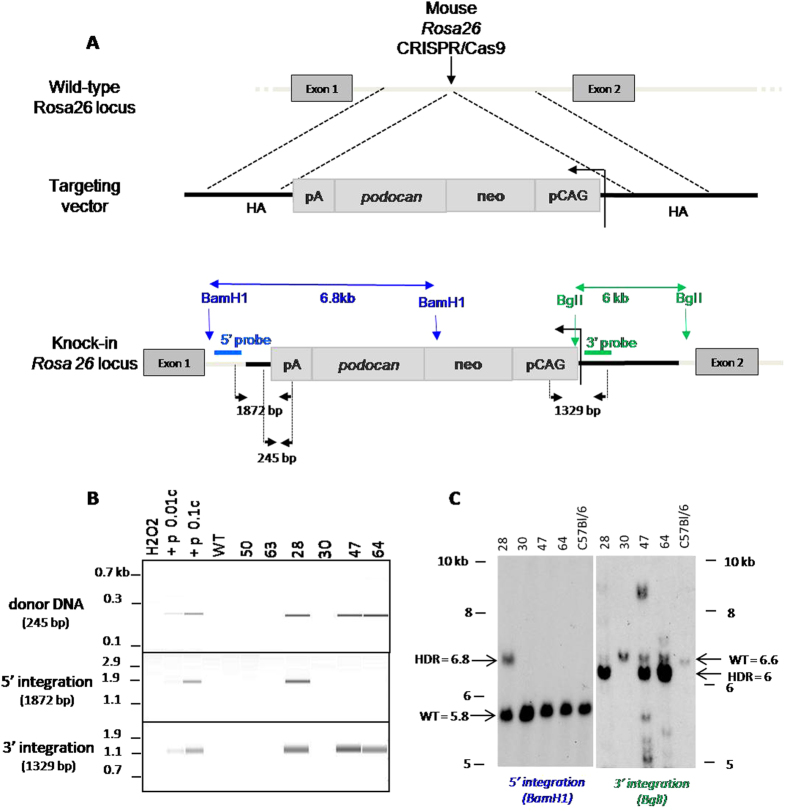
Targeted integration of a podocan instead of GFP cassette into the mouse *Rosa26* locus. (**A, upper**) The mouse *Rosa26* locus. The site of CRISPR/Cas9 action (vertical arrow) and the targeting vector with the expression cassette (7.1 kb) and the 5′ and 3′ homology arms (HA) (5′ and 3′ 1034 bp and 4288 bp, respectively) are indicated. **(A**, lower) Expression cassette integration. Genomic DNA was PCR amplified with primers in flanking regions of the 5′ and 3′ homology arms. The integration of the expression cassette was also analyzed using a primer situated in the 5′ HA arm and in the polyA. BamHI or BglI sites used for Southern blot are indicated as well as probes used and the predicted size of the bands for faithful HDR (6.8 or 6 kb for BamHI or BglI digestions, respectively). (**B**) PCR analysis. Capillary gels show the expression cassette (upper gel) with an expected band of 245 bp in fetuses N° 28, 47 and 64. Analysis of the 5′ and 3′ extremities of the expression cassette (middle gel and lower gels, respectively) revealed that fetus N° 28 showed the expected band of 1872 bp and 1329 bp whereas the other fetuses N° 47 and 64 only showed the 3′ band of 1329 bp, suggesting complete and incomplete HDR, respectively. Podocan- E15 fetuses N° 50, 63 and 30 are negative controls. Positive controls were 0.01 or 0.1 copies of a plasmid containing the genomic flanking sequences and the expression cassette (p0.01c and p0.1c). (**C**) Southern blot analysis. DNA from podocan+ fetuses N° 28, 47 and 64 and one podocan- N° 30 were digested with BamHI or EcoRI. For the 5′ extremity, only fetus 28 showed a band of the expected size (6.8 kb) for HDR and a band of 5.8 kb found in WT alleles that was also found in all other fetuses and a C57Bl/6 WT animal, indicating absence or incomplete HDR. For the 3′ extremity, fetuses 28, 47 and 64 showed bands of the expected size for HDR (6 kb) whereas fetus 30 and the C57Bl/6 WT animal showed a wild type band (6.6 kb).

To extend the results obtained with Cas9 protein in rats to mice, we microinjected mouse zygotes using the same technique used for rats. The sgRNA targeted intron 1 in the mouse *Rosa26* locus and the donor DNA used was a linear longer fragment (12.5 kb, 2 ng/μl) compared to the rat one, aimed at introducing an expression cassette for CAG-podocan coding sequences (Fig. 3A). Preliminary experiments showed that Cas9/sgRNA protein complex microinjected at 6 and 3 μM resulted in embryo mortality (data not shown) whereas microinjections at 0.3 or 1.5 μM resulted in acceptable embryo survival and day 13 fetus development comparable to that observed with Cas9 mRNA at 20 ng/μl (Table 2) and to those obtained with other constructs (data not shown). Day 13 fetuses that had been microinjected with Cas9 protein at 0.3 μM exhibited HDR in 2.85% of transferred embryos (16.7% of analyzed fetuses) and NHEJ in 7.1% of transferred embryos (42% of analyzed fetuses) (Table 2). Both fetuses (N° 47 and 64) obtained with Cas9 protein at 0.3 μM showed PCR positive expression cassette, PCR positive for 3′ integration but not for the 5′ integration PCR (Fig. 3B) and Southern blot bands of expected size using a 3′ probe but not a 5′ probe (Fig. 3C), and thus represent incomplete HDR. Cas9 protein microinjected at 1.5 μM resulted in HDR in 1.5% of transferred eggs (16.7% of analyzed fetuses) and NHEJ in 9.1% of transferred eggs (100% of analyzed fetuses). In one fetus (N° 28) HDR was evidenced by positive expression cassette and flanking PCRs of the expected size (Fig. 3B) and Southern blot with 5′ and 3′ bands of the expected size (Fig. 3C). Fetuses that were microinjected with Cas9 mRNA/sgRNA resulted in no HDR and in NHEJ in 13.4% of transferred eggs (94% of analyzed fetus) (Table 2). One fetus showed positive podocan PCRs but not flanking PCRs and thus represents a random genomic insertion of the donor DNA (Table 2). Thus, for the mouse *Rosa26* locus Cas9 protein was more efficient in generating HDR than Cas9 mRNA, whereas for NHEJ induced mutations Cas9 protein and mRNA were comparable.

### Microinjection of Cas9 proteins or mRNA targeting the *Foxp3* locus into rat zygotes to obtain HDR

**Figure 4:**
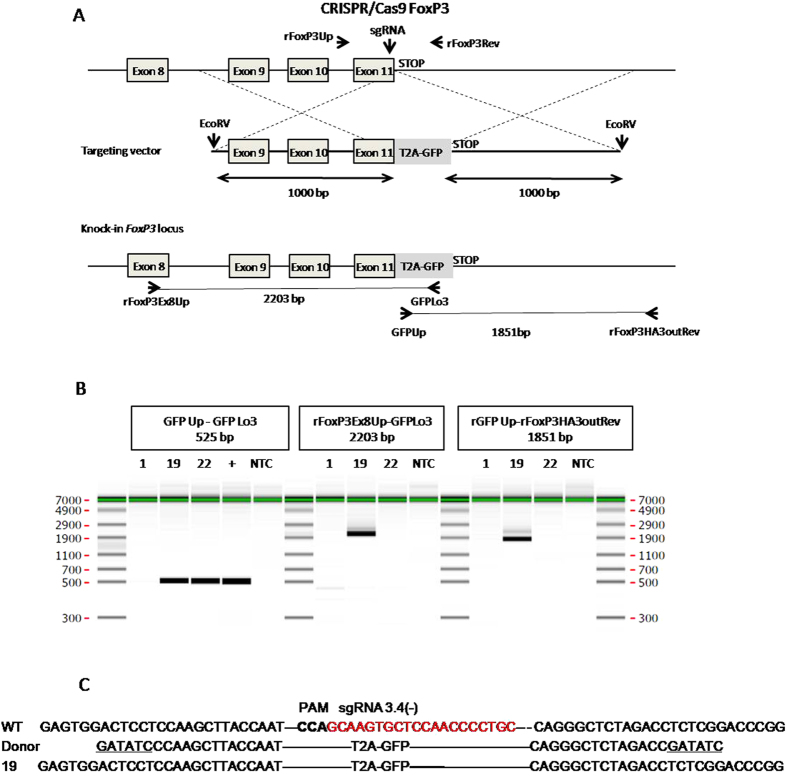
Targeted integration of a GFP cassette into the rat *FoxP3* locus. (**A, upper**) Schematic representation of the rat *FoxP3* locus. The site of CRISPR/Cas9 nuclease action (vertical arrow) and of the targeting vector with the T2A-GFP cassette (783 bp) and the 5′ and 3′ homology arms (HA) (1000 bp each) are indicated. The homology arms are contiguous to the CRISPR/Cas9 nuclease cleavage point. EcoRV restriction sites are indicated. (A, lower) Diagram showing schematic representation of the GFP cassette integration. For PCR analysis, genomic DNA was PCR amplified with primers situated in the T2A-GFP sequence for detection of the transgene (GFPUp and GFPLo3), for the 5′ side upstream of the 5′HA (rFoxP3Ex8Up) and in the GFP cDNA (GFPLo3), and for the 3′ side: in the GFP cDNA (GFPUp) and downstream of the 3′ HA (rFoxP3HA3outRev). The position of each primer and the corresponding expected size of PCR products are indicated on the schematic knock-in *FoxP3* locus. (**B**) PCR analysis. Capillary gels show the presence of the donor DNA (left gel) with the presence of a specific band of 525 bp in two fetuses (N° 19 and 22). Analysis of the 5′ and the 3′extremities (middle and right gels) of the expression cassette integration into *FoxP3* locus of the two GFP+ founders showed in one of them (N° 19) expected bands of 2203 bp using the 5′ pair of primers (rFoxP3Ex8Up - GFPLo3) and of 1851 bp using the 3′ pair of primers (GFPUp - rFoxP3HA3outRev), suggesting faithful HDR. The other GFP+ fetus (N° 22) did not show these bands, indicating a random integration. A GFP- E15 fetus (N° 1) is shown as negative control. + is the positive control for PCR using the donor plasmid. NTC indicates PCR in the absence of DNA. (**C**) Analysis of flanking sequences. Comparison of sequences at the 5′ and 3′ junctions with wild-type genomic DNA and donor DNA sequences of the knock-in founder (N° 19). In donor DNA, the presence of the EcoRV site is indicated (underlined). On the WT allele, the sgRNA is indicated in red (PAM in bold).

To extend and confirm the results obtained in the *Rosa26* locus, we targeted the rat *Foxp3* locus using a sgRNA for a sequence of the last gene exon (exon 11) situated just 5′ of the stop codon (Fig. 3A). Rat zygotes were microinjected in the pronuclei and the cytoplasm using a mixture of either a Cas9/sgRNA protein complex (equimolar 3 μM) or mRNA/sgRNA along with donor DNA. The donor DNA contained a 5′ homology arm of 1 Kb comprising *Foxp3* exons 9–11 and the respective introns, a T2A-GFP-stop codon sequence and a 3′ homology of 1 Kb arm comprising *Foxp3* 3′ untranslated sequences (Fig. 4A). Day 15 embryo DNA was analyzed by PCR of the GFP coding sequence while GFP expression was not analyzed since it is under the control of the *Foxp3* promoter and it is not expected at this early time point in ontogeny. HDR and NHEJ were analyzed by PCR and sequencing of the amplicons of 5′ and 3′ insertion extremities of the donor DNA and of the exon 11 targeted sequence, respectively. Cas9 protein was not toxic and resulted in a HDR rate of 0.8% of transferred eggs (3.44% of day 15 embryos, n = 29) and in a NHEJ rate of 1.6% of transferred eggs (6.9% of day 15 embryos) (Table 3 and Fig. 4B). In contrast, Cas9 mRNA did not result in the generation of HDR and resulted in NHEJ in 2.6% of transferred eggs (7.7% of day 15 embryos analyzed n = 52) (Table 3 and Fig. 4B). For both Cas9 protein and mRNA, animals with random insertion of the donor DNA were identified with similar frequencies (0.8% and 0.7%, respectively) (Table 3). Sequencing of the amplicons generated with primers flanking the 5′ and 3′ insertion extremities showed the exact wild type sequences as well as internal T2A and GFP indicating faithful HDR (Fig. 4C). There were no mosaic animals among any of the Cas9 protein or mRNA mutated animals (data not shown).

Thus, for the *Foxp3* locus also, Cas9 protein was more efficient than Cas9 mRNA at generating HDR using a long DNA donor fragment with NHEJ rates comparable.

### Microinjection of Cas9 proteins or mRNA targeting the *Anks3* locus along with ssODNs into rat zygotes to obtain HDR

**Figure 5:**
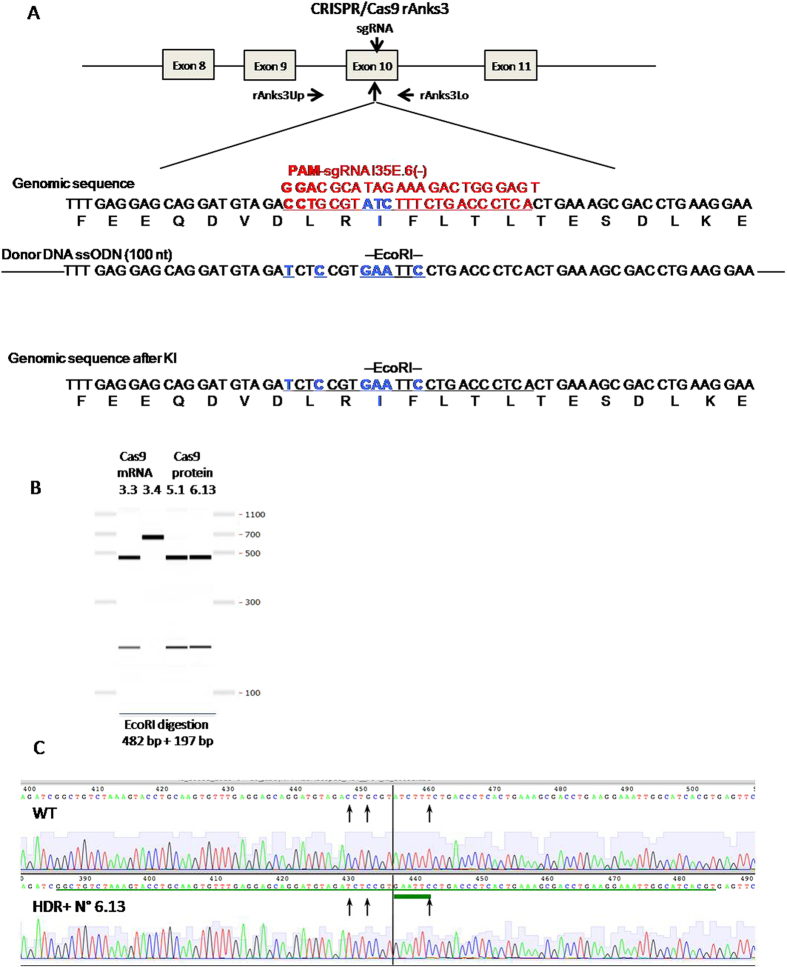
Targeted integration of a point mutation into the rat *Anks3* locus. (**A, upper**) Schematic representation of the rat *Anks3* locus. The site of CRISPR/Cas9 nuclease action (vertical arrow) and of the targeting site of the sgRNA used (in red, PAM in red bold) are indicated. Genomic DNA was PCR amplified with primers situated upstream (rAnks3Up) and downstream (rAnks3Lo) of the sgRNA site and followed by EcoRI restriction. The ssODN (100 nucleotides) carries the mutation ATC/GAA and others mutations on the sgRNA (in blue) to cancel the CRISPR/Cas9 nuclease cleavage after HDR. EcoRI restriction site is indicated (underlined). (A, lower) Diagram showing schematic representation of mutation integration. The mutations are labeled in blue and the EcoRI site is underlined. (**B**) PCR analysis. Capillary gels show the analysis of the integration into *Anks3* locus. PCR products of the exon 10 (rAnks3Up-Lo) were digested with EcoRI. Three fetuses (N° 3.3, 5.1 and 6.13) showed digestion by EcoRI with bands of the expected size (482 bp and 197 bp) for HDR, and were homozygous for HDR (N° 3.3 with incomplete HDRs with indels in the extremities but conserved EcoRI site), explaining the absence of a wild-type band (679 bp). A rat with no HDR (3.4) shows a band of 679 bp that is not digested by EcoRI and is shown as negative control. The microinjection of Cas9 mRNA (for animal 3.3) or Cas9 protein (for animals 5.1 and 6.13) are above each animal. (**C**) Analysis of knock-in sequences. Comparison of sequences at the mutation site between wild-type genomic DNA and DNA sequences of one representative HDR+ homozygous founder (6.13). In HDR + DNA, the presence of the ssODN (green underlined), the ATC/GAA mutation (green thick underlined) and sgRNA mutations site (vertical arrows) are indicated.

Since for the *Rosa26* loci the increase in HDR using Cas9 protein could be the result of the associated increase of NHEJ that was also observed, we decided to decrease the concentration of sgRNA to suboptimal conditions for DNA cleavage and analyze HDR efficiency under these conditions. For this, we used a sgRNA for exon 10 of the rat *Anks3* gene (Fig. 5A) that showed high NHEJ activity with Cas9 mRNA and we used the Cas9/sgRNA protein complex at the optimal Cas9 protein concentration and a lower concentration of the sgRNA (3 μM/1.5 μM) compared with the previous two loci. For the *Anks3* locus, the objective was to introduce a point mutation (ATC to GAA or I35E) and for this we microinjected ssODNs (7.5 ng/μl) instead of long DNA donor fragments. The donor DNA also contained silent mutations that created an EcoRI site to facilitate the detection of HDR. As far as we know, the use of Cas9 protein and ssODNs has not been used before in mammals since the only previous publication looked at zebra fish^15^.

Pups born after microinjection were analyzed by PCR of exon 10 encompassing the whole donor sequence and extending into the genomic unmodified sequence (Table 4 and Fig. 5B). Animals generated with Cas9 protein showed HDR in 4 animals, 2.6% of transferred zygotes (18.2% of born animals). In 2 out of these 4 animals both alleles were HDR as evidenced by PCRs with bands of the expected size that were cleaved by EcoRI (Table 4 and Fig. 5B) and by sequencing of these PCR products (Fig. 5C and data not shown). All of these HDR were complete, with no indels in the extremities (Fig. 5C and data not shown). In contrast, only one animal generated with Cas9 mRNA (N° 3.3) showed HDR and this was not faithful since indels were observed in both extremities of the inserted sequence but that they did not affect the EcoRI site (Table 4, Fig. 5C and data not shown). The rate of NHEJ was higher in animals generated using Cas9 mRNA since 6.7% of transferred zygotes (41.2% of born animals) displayed NHEJ mutations whereas animals generated with Cas9 protein (n = 22) showed 2.6% NHEJ in transferred zygotes (18.2% of born animals) (Table 3). Mosaicism was observed in 66.5% of Cas9 protein and 25% of Cas9 mRNA injected animals. Thus, Cas9 protein was also more efficient at generating HDR and resulted in higher degree of mosaicism than Cas9 mRNA for a locus in which NHEJ was high and in favor of Cas9 mRNA.

## Discussion

The generation of genetically-modified animals has been greatly improved recently by the direct microinjected in one-cell stage embryos of gene-specific nucleases, such as ZFNs, TALENs and CRISPRs-Cas9^2^,^3^,^5^. This technique has made it possible to generate targeted knockout and knockin genes in species other than mice, i.e. species which do not have the advantage of the existence of robust ES cells. In these species, the generation of genetically modified animals was based on inefficient, cumbersome techniques, such as random mutagenesis, random transgene integration by DNA microinjection and cloning by somatic nuclear transfer. Even in the mouse, for which there are robust ES cells-based technologies, genome editing is now being increasingly carried out using gene-specific nucleases since they represent an advantage in terms of both time and cost^2^. Despite these advances, the efficiency in the generation of genetically-modified animals, particularly knockin, using gene-specific nucleases, is still low. Therefore, it would seem advantageous to increase the efficiency of DNA cleavage and/or donor DNA integration.

The use of protein nucleases could increase the rate of DNA cleavage and/or of HDR efficacy since they can cleave the DNA faster than when delivered as mRNA and could thus increase the availability of donor DNA for incorporation into the cleavage point. Cas9 protein microinjected in zebrafish embryos induces NHEJ faster compared to mRNA^16^, whether this can be confirmed in mammal zygotes still needs to be analyzed. Cas9 protein shows increased HDR of short sequences using ssODNs in zebrafish compared to mRNA^15^. Two other studies have used Cas9 protein for gene invalidation by NHEJ (no HDR reported), they show comparable mutation rates to mRNA in zebrafish and mice^16^ but there was no comparison to mRNA in *C. elegans*^17^. This present work applies Cas9 protein to rats for the first time and in addition, obtains HDR of a large DNA donor fragment in rats and mice. This was not explained by higher frequencies of NHEJ since we observed that with Cas9 protein or Cas9 mRNA using the same sgRNA these frequencies varied; in the rat *Rosa26* locus Cas9 protein was more efficient than Cas9 mRNA, but in the mouse *Rosa26* locus, as well as in the rat *Foxp3,* there was comparable efficiency, and for the *Anks3* locus the NHEJ frequencies for Cas9 protein were lower than those for Cas9 mRNA. Thus, the differences in the concentration of sgRNA used when Cas9 protein or mRNA were microinjected (higher for the former) cannot explain the observed differences in NHEJ. As for HDR, Cas9 protein was more efficient than Cas9 mRNA in every case when using both long donor DNA fragments and ssODNs. In fact, several of the HDR-mutated rats and mice could not have been generated without the use of Cas9 protein. When using ssODNs, Cas9 mRNA did generate HDR, but showed indels in the extremities of the incorporated sequences and this introduced out of frame mutations when the objective was to introduce a single amino acid mutation. In contrast, all the HDRs obtained using Cas9 protein and ssODNs were without indels. The better HDR results obtained with Cas9 protein using both the long and short donor DNA suggest that faster HDR kinetics could avoid donor DNA degradation before genomic integration.

The concentration of the donor DNA microinjected (2 ng/μl) into the pronucleous and immediately after into the cytoplasm is the one that we have previously used as optimal when performing HDR using TALENs^1^,^18^. This concentrations is lower compared to those used in other studies using microinjection into the pronucleous of long double stranded DNA as donors in the rat [4 ng/μl^10^,^11^,^12^ or 10 ng/μl^19^], as well as in mice [10 ng/μl^20^] but in our technical conditions higher concentration of DNA results in excessively high embryo toxicity. Higher concentrations of long double stranded donor DNA were optimal when microinjected into the cytoplasm but the optimal for pronuclear microinjection were much lower (200 as compared to 10 ng/μl, respectively)^14^. The concentration of ssODNs we used were higher (7.5 ng/μl) but the differences in toxicity with long double stranded donor DNAs can be explained by intrinsic differences of both DNA forms.

The long donor DNAs were used in a linear form because we have shown that two different DNA donors, including the one for CAG-GFP for the rat *Rosa26* locus, generated HDR when using TALENs only when in a linear form and not in a circular one^1^. Previous publications in mice and rats showed that in some of them both linear and circular DNA generated HDR^21^,^22^,^23^,^24^ whereas others showed that only the linear one was successful^25^. Furthermore, although the circular form has been described as reducing genome integration as compared to the linear form^26^, in our conditions, both the linear and circular forms generated a comparable proportion of random integration^1^.

Mutated animals generated using nucleases expressed by nucleic acids frequently exhibit mosaicism^1^,^2^, probably due to the initial action of nucleases after the one-cell stage embryo stage and/or the persistent action of nucleases beyond the one-cell zygote stage, which re-cleave alleles that were repaired faithfully by NHEJ at the one-cell stage. Thus, a potential advantage of the use of protein nucleases could be a lower percentage of mosaic animals, since cleavage could begin in the one-cell stage embryos. However, if the half-life of the protein goes beyond the one-cell stage embryo stage, then its action could be a source of mosaicism. The results in the present study show that some Cas9 protein obtained animals are mosaic and the frequency is comparable to that using Cas9 mRNA, suggesting that this is possibly due to the half-life of Cas9 and its action being beyond the one-cell embryo stage. Previous analysis of *C. elegans* mutants obtained using Cas9 protein showed the presence of mosaic animals and although this work did not include a comparison with the use of mRNA, it does show that mosaicism was not eliminated^17^. Mosaicism using Cas9 protein could eventually be reduced using a lower protein concentration, thus favoring its action at the one-cell embryo stage.

Although GFP expression in tissues of the rat *Rosa26* HDR fetus harboring a CAG-GFP expression cassette was not analyzed, we have previously shown that this same cassette introduced by HDR in the rat *Rosa26* locus using TALEN mRNA resulted in fetuses that are brightly and uniformly GFP positive and correlated with widespread tissue expression on adult animals^1^.

To the best of our knowledge this is the first time that TALEN proteins have been used to obtain genetically-modified animals. Despite TALEN proteins performing less well than mRNA, the highest concentration microinjected still maintained embryo survival and while using higher concentrations might have obtained higher mutation rates, this was technically not possible due to TALEN protein precipitation. We cannot exclude other TALEN purification techniques obtaining higher concentrations. Nevertheless, the ease of generation of sgRNAs and the universal function of Cas9 along with the superior results in transgenesis clearly point to the CRISPR-Cas9 system being more versatile than TALENs. At the same time, for some sequences for which TALENs are already available, such as the ones targeted here in the rat *Rosa26* locus, the rate of embryos with NHEJ or HDR with the TALEN mRNA (3.3%)^1^ was comparable to that of the CRISPRs/Cas9 protein system (3.5% at 3 μM of Cas9 protein).

Although ZFNs^27^, TALENs^28^ and Cas9^29^ fused to cell-penetrating peptides or anionic proteins^30^ have been delivered as proteins to cells *in vitro*, and resulted in comparable or superior efficacies to mRNA for NHEJ and HDR using ssODNs^30^, these techniques are not applicable to zygotes due to the zona pellucida barrier.

The rat is a widely used experimental model that is gaining popularity due to the generation of many new genetically engineered models using gene-specific nucleases^5^. The increase in efficiency and refinement of the genome editing and transgenic technologies through gene-specific nucleases should increase the use of rats as an alternative or complement to other experimental species.

In conclusion, we have shown that the use of Cas9 protein increased NHEJ and/or HDR efficiency depending on the locus when compared to injection of Cas9 coding mRNA. In contrast, injection of TALEN proteins was less efficient than injection of coding mRNA. Combined with the other numerous advantages of CRISPR/Cas9 technology in the generation of genetically-modified animals, the use of Cas9 protein further increases the efficiency and applications in genome editing.

## Methods

### Animals

The rats used in this work were from the Sprague-Dawley (SD/Crl) strain (Charles River, L’Arbresle, France). The mice were from the C57Bl6/N strain (Janvier Labs, Le Genest St Isle, France). The study was approved by the Ethics Committee on Animal Experimentation of the Pays de la Loire Region, France, in accordance with the guidelines from the French National Research Council for the Care and Use of Laboratory Animals (Permit Numbers: CEEA-PdL-2011-45). All efforts were made to minimize suffering.

### Plasmid construction for TALEN expression in *E.coli*

TALE nucleases for rat *Rosa26* locus (Mar. 2012 (RGSC 5.0/rn5) genome Assembly. ROSA26 (chr4: 208314007-208314058)).


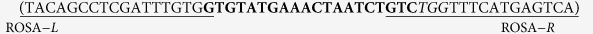


recognize a sequence in intron 1 and have been already described^1^. These sequences were subcloned in the bacterial expression vector pSKB3 (kind gift from Dr. Stephen K Burley) using an In Fusion cloning kit (Clontech) (see sequences in Supplementary Materials). The pSKB3-ROSA constructs were expressed in the *E. coli* Rosetta (DE3) pLysS (Novagen) strain. One kanamycine resistant clone was inoculated in 50 mL of LB containing Kanamycine (50 μg/mL) and 2% glucose and was grown overnight at 37 °C at 200 rpm. Next morning, 2 L Erlenmeyer flasks containing 500 mL of LB supplemented with Kanamycin sulfate (50 μg/mL) and 1% glucose were inoculated at OD_600_ = 0, 1 and bacteria were grown at 37 °C, 200 rpm for about 3 h to reach OD_600_ = 1.2 to 1.4. IPTG was added to a final concentration of 0.2 mM and induction was done for 22 h at 16 °C, shaking to 200 rpm. Cells were harvested and washed with PBS before freezing at −80 °C. Cells were sonicated in 20 mL of lysis Buffer (25 mM Tris-OAc pH 8.0, 300 mM NaCl, Protease Inibitor Ultra -EDTA (Roche), 1 mM PMSF) and lysate was centrifugated at 92,600 g at 4 °C for 1h30 in a 70TI rotor (Beckman). Supernatant was loaded on 2 mL of HIS-select-HF Nickel affinity gel (SIGMA), extensively washed with 25 mM Tris-HCl pH 8.0, 300 mM NaCl followed by 8 mL of 25 mM Tris-OAc pH 8.0, 300 mM NaCl, 10 mM Imidazole and eluted in 8 mL of 25 mM Tris-OAc pH 8.0, 300 mM NaCl, 250 mM Imidazole. Proteins were precipitated with 20% ammonium sulfate (adding 0.85 g to 8 mL) and centrifuged 30 min at 4 °C at 13,000 g. Precipitated proteins were solubilized overnight on ice in 300 μL of 25 mM Tris-OAc pH 8.0, NaCl 300 mM, EDTA 1 mM, TCEP 1 mM, protease inhibitor complete (Roche). Protein was further purified on a Superdex 200 GL 10/300 (GE-Healthcare) using an AKTA purifier 10 against 25 mM Tris-OAc pH 8.0, 300 mM NaCl, 1 mM EDTA. Protein was concentrated to 21 μM on microcon 10 kDa (Millipore), supplemented with 10% glycerol and flash frozen in nitrogen before storage at −80 °C.

### Plasmid construction for *S. pyogenes* Cas9-3NLS nuclease expression in *E. coli*

Plasmid pMJ806 was obtained from Addgene (http://www.addgene.org/39312) and since 2xNLS were more efficient at targeting Cas9 to the nucleus than 1xNLS^31^ we introduced 3xNLS by ligation independent cloning (Gibson Assembly, New England Biolabs) (see coding sequences in Supplementary Materials).

### Expression and purification of *S. pyogenes* Cas9-3NLS nuclease

Expression and purification of *S. pyogenes* Cas9 protein was adapted from Jinek *et al.*^32^. The main modification consisted in carrying out protein expression using a 3 L computer-controlled bioreactor (Lambda, CZ) using growth conditions as described in Marisch *et al.*^33^. Briefly, 2, 5 mL of a pre-culture of Rosetta2 bacterial strain transformed with pMJ806-3NLS construct at OD600 = 0.8 was inoculated in a semi synthetic batch medium (1.5 L), containing for the present set up 4.5 g KH_2_PO_4_, 9 g K_2_HPO_4_.3H_2_O, 25 g of glucose.H_2_O, 2.1 g of sodium citrate (trisodium salt. 2H_2_O), 0.84 g MgSO_4_.7H_2_O, 0.168 g CaCl_2_.2H2O, 1.26 g of yeast extract, 420 μL of trace element solution prepared as described in Marisch *et al.*^33^, and 50 mg/L of kanamycin sulfate. The pH was maintained at 7.0 by addition of 14% NH_4_OH. Foaming was automatically suppressed by addition of Antifoam C emulsion (Sigma) as the modulation of conductance was detected with the foam formation (maximum 0.5 mL/L). Bacteria in batch medium were grown for 11 h at 37 °C with 5 Hz vertical stirring and 4 L/min air flow (DO600 = 3.5). Exponential feeding with a flow rate allowing addition of 600 mL of fed batch medium in 42 h according to exponential growth algorithm X = X_0_.e^μt^ (with μ = 0.1 h^−1^) was then started overnight. Composition of the fed batch medium was the following: 3 g/L of KH_2_PO_4_, 6 g/L of K_2_HPO_4_.3H_2_O, 21 g/L of sodium citrate (trisodium salt. 2H_2_O), 9 g/L MgSO_4_.7H_2_O, 1.74 g/L of CaCl_2_.2H2O, 4.3 mL/L of trace element solution, 258 g/L of glucose. H_2_O and 50 mg/L of kanamycin sulfate. After 18 h of feeding, (OD_600_ = 16.2) temperature was decreased to 16 °C and protein expression was induced by a first pulse of IPTG (65 μM) followed by a second pulse 30 min later (+0.325 mM). 0.2 mM IPTG was also added to the feed medium. Feeding was continued for 23 h to reach 2 L with OD_600_ = 32 and 118 g of bacterial pellets. Protein purification was then carried out as described in Jinek *et al.*^32^.

### Guide RNA plasmid construction

The sgRNA for rat *Rosa26* targets a sequence (GTGTATGAAACTAATCTGTC*TGG*) (*P*AM in italics) in intron 1, in bold in the TALEN Rosa26 sequence depicted above. The sgRNA for mouse *Rosa26* targets a sequence (GAAGATGGGCGGGAGTCTTC*TGG*) (*P*AM in italics) in intron 1. The sgRNA for rat *Foxp3* targets a sequence (GCAGGGGTTGGAGCACTTGC*TGG*, PAM in italics) in 3′ end of the last exon (exon 11), just before the stop codon. The sgRNA for rat *Anks3* targets a sequence (*GGA*CGCATAGAAAGACTGGGAGT, PAM in italics) in exon 10.

sgRNAs were cloned in MLM3636 and DR274 derived vectors (http://www.addgene.org/43860/ and http://www.addgene.org/42250/) for U6 and T7 transcription and used in cell cultures or for one-cell embryo injections, respectively. For cellular experiments and mRNA injection, human codon-optimized Cas9-expression vector (hCas9, http://www.addgene.org/41815/) was used.

### *In vitro* transcription and purification of mRNA

sgRNAs were transcribed using the DraI-digested sgRNA expression vectors DR274 as templates and the T7 high yield kit (New England Biolabs). Following transcription DNase I (New England Biolabs) treatment was carried out and sgRNAs were purified using EZNA microelute RNA Clean-UP column (OMEGA Biotek).

The Cas9 mRNA was transcribed using Pme1-digested Cas9 expression vector (http://www.addgene.org/43861/) and the mMESSAGE mMACHINE T7 ULTRA kit (Life Technologies). Following transcription, the poly(A) tailing reaction and DNase I treatment were performed according to the manufacturer’s instructions. The Cas9- encoding mRNA was then purified using Megaclear kit (Life Technologies).

### Rosa26 DNA *in vitro* cleavage assay using TALEN and Cas9 proteins

The rat *Rosa26* locus was PCR amplified (see sequences in supplementary Tables) from rat C6 line cells using specific primers. Amplified DNA was digested in the presence of TALEN ROSA-L and ROSA-R proteins or of Cas9 protein sgRNA complexes at various concentrations. Samples were analyzed on an agarose gel (2.5% TBE 0.5X) with SYBR green I post-straining.

### TALEN or Cas9 mRNA activities in rat cells

The TALEN or sgRNA and Cas9 mRNAs were transfected in rat C6 cells fibroblasts using RNA MessengerMax reagent (Life Technologies). Cells were grown in 24-well dishes and collected 48 h after treatment. Cells were lysed and the genomic region encompassing the nuclease target sites was directly amplified with specific primers and fusion polymerase (New England Biolabs). T7 Endonuclease I assays were performed and estimated NHEJ frequencies were calculated as previously described^34^.

### DNA donors

The rat *Rosa26* DNA donor (4.7 kb) contained two 800 bp homologous arms sequences of the rat *Rosa26* locus, immediately contiguous to the site of DNA cleavage (intron 1) by both TALENs and Cas9, flanking an expression cassette with the CAG promoter-eGFP cDNA-BGHpA (Fig. 2A) as has already been described in detail^1^. The mouse *Rosa26* DNA donor (12.5 kb) contained two homologous arm sequences of the mouse *Rosa26* locus of 1034 bp and 4288 bp (5 and 3′, respectively), immediately contiguous to the site of DNA cleavage (intron 1) by Cas9, flanking an expression cassette with the CAG promoter-podocan gene (Fig. 3A). The rat *Foxp3* DNA donor (2.8 kb) contained two 1 kb homologous arm sequences of the rat Foxp3 locus, immediately contiguous to the site of DNA cleavage by Cas9 (exon 11, upstream of the stop codon), flanking a cassette containing a T2A self-splicing sequence and eGFP (Fig. 4A). The rat *Anks3* DNA donor (100 bp) contained two homology arms of 51 and 46 bp and in between a sequence introducing a ATC to GAA mutation in position I35 and T to C mutation to obtain a conserved mutation that generates an EcoRI restriction site for the ease of analysis of HDR (Fig. 5A).

### Microinjection of rat and mouse one-cell embryos

Cas9 protein and sgRNA at different concentrations indicated in the results section for each locus (for example, 3 μM of Cas9 protein corresponded to 475 ng/μl and 3 μM of sgRNA to 100 ng/μl) were incubated at room temperature for 10 min to allow formation of ribonucleoprotein complexes and then were kept at 4 °C until microinjection.

For all rat loci Cas9 mRNA was microinjected at 50 ng/μl and sgRNA at 10 ng/μl and for mouse Cas9 mRNA at 20 ng/μl and sgRNA at 10 ng/μl, concentrations close to those previously described as successfully generating mutated rats^10^,^11^,^12^ and mice^13^,^14^. TALEN monomer proteins were also microinjected at different concentrations (4 or 10 μM final concentration for both monomers). Mixtures of linear donor DNA (2 ng/μl) or ssODNS (7.5 ng/μl) were mixed with Cas9 (and sgRNA) or TALENs either as protein or mRNA immediately before use, briefly centrifuged at 17,950 g (10 min at 4 °C) and kept on ice during microinjection. Fertilized 1-cell stage embryo collection and sequential microinjection into the male pronucleus and into the cytoplasm have previously been described in detail^1^. Microinjected embryos were maintained under 5% CO_2_ at 37 °C 3 h. Surviving embryos were then implanted immediately in the oviduct of pseudo-pregnant females (0.5 days post coitum) and allowed to develop until embryonic day 15 for rats and day 13 for mice, or until normal delivery.

### Analysis of GFP expression

GFP expression was monitored in E15 fetuses and pups using a Dark Reader® Spot Lamp (Clare Chemical Research, Inc.).

### Analysis of NHEJ events

Briefly, DNA targeted regions were PCR amplified with a high-fidelity polymerase (Herculase II fusion polymerase) using specific primers (supplementary Table 1). Mutations were analyzed using the T7 endonuclease I assay and direct sequencing of PCR products as previously described in detail^1^,^6^.

### Analysis of targeted HDR and random integration of DNA donor sequences

DNA from embryos or neonates was extracted from tail biopsy following treatment with Proteinase K as previously described^1^,^6^. We first identified animals carrying a transgene (GFP or others) by PCR amplification using a primer for the transgene (supplementary Table 1). Each transgene positive rat was then analyzed with PCRs flanking the donor DNA sequence using primers in the genomic sequences situated outside the homology arms and others in the inserted sequence (supplementary Table 1). These PCR fragments were then sequenced to detect genomic sequences flanking the donor DNA homology arms. For rat *Rosa26*, Southern-blots were also carried out on genomic DNA digested by EcoRI. After digestion, the DNA was separated by agarose gel electrophoresis, transferred to a nylon membrane (Hybond XL, GE Healthcare), hybridized with a DNA probe labeled with α^32^P-dCTP, washed and subjected to autoradiography.

## Additional Information

**How to cite this article**: Ménoret, S. *et al.* Homology-directed repair in rat zygotes using Cas9 and TALEN engineered proteins. *Sci. Rep.* **5**, 14410; doi: 10.1038/srep14410 (2015).

## Supplementary Material

Supplementary Information 1

## Figures and Tables

**Table 1:** Modification of the rat *Rosa26* locus using Cas9 or TALEN proteins.

Form Cas9 or TALEN	ConcentrationCas9/sgRNA	N°. injectedeggs(% viable)	N°.transferredeggs	N° E15(%)^a^	N°. UVGFP+(%)^a^	N°. PCRGFP+(%)^a^ Tg	N°. HDR+ (%)^a^;(%)^b^ HDR	N°. of indel+ (%)^a^(%)^b^ NHEJ
Rosa26 Cas9 protein*	0.3 μM/0.3 μM	162 (79)	120	52 (43.3)	3 (2.5)	6 (5.0)	0 (0)	0 (0)
	3 μM/3 μM	160 (75.6)	117	57 (48.7)	2 (1.7)	0 (0)	2 (1.7); (3.5)	13 (11.1) (22.8)
	6 μM/6 μM	188 (76)	143	23 (16.1)	1 (0.70)	0 (0)	1 (0.7); (4.35)	3 (2.1) (13)
Rosa26 Cas9 mRNA*	50 ng μl/10 ng μl	208 (82.2)	171	53 (31)	1 (0.6)	1 (0.6)	0 (0)	4 (2.35) (7.55)
Rosa26 TALEN proteins**	4 μM	184 (75)	130	43 (33.1)	1 (0.8)	1 (0.8)	0 (0)	0 (0)
	10 μM	144 (81.2)	117	28 (24)	0 (0)	0 (0)	0 (0)	3 (2.6) (10.7)
Rosa26 TALEN mRNA 1***	10 ng/μl	161 (80.8)	119	11 (13)	2 (1.7)	0 (0)	2 (1.7)	5 (4.17) (45.45)

*Cas9 protein at different concentrations: sgRNA preformed equimolar complexes or Cas9 mRNA mixed with sgRNA were microinjected at the indicated concentrations along with linearized donor DNA at 2 ng/μl. **both TALEN monomers microinjected along with linearized donor DNA at 2 ng/μl. ***both TALEN monomers microinjected along with linearized donor DNA at 2 ng/μl. ^a^(% of transferred embryos). ^b^(% of E15 fetus). Tg, transgenics by random integration; HDR, homology-directed repair; NHEJ, non-homologous end joining; E15: embryos at day 15. ^1^results of HDR for TALEN mRNA already reported.

**Table 2:** Modification of the mouse *Rosa26* locus using Cas9 proteins.

Form Cas9	ConcentrationCas9/sgRNA	N°.injected eggs(% viable)	N°.transferredeggs	N° E13(%)^a^	N°. internalPCR+(%)^a^ Tg	N°. HDR/analyzed(%)^a^ (%)^b^ HDR	N°. of indel+/analyzed (%)^a^(%)^b^ NHEJ
Rosa26 Cas9 protein	0.3 μM/0.6 μM	114 (61%)	70	14 (20%)	0	2/12* (2.85) (16.7)	5/12 (7.1) (42)
Rosa26 Cas9 protein	1.5 μM/3 μM	118 (56%)	66	7 (11%)	1 (1.5)	1/6 (1.5) (16.7)	6/6 (9.1) (100)
Rosa26 Cas9 mRNA	20 ng μl/10 ng μl	163 (78%)	127	21 (16%)	0	0	17/18 (13.4) (94)

Cas9 protein sgRNA preformed equimolar complexes or Cas9 mRNA mixed with sgRNA were microinjected at the indicated concentrations along with linearized donor DNA at 2 ng/μl. ^a^(% of transferred embryos). ^b^(% of day 13 analyzed fetuses). Tg, transgenics by random integration; HDR, homology-directed repair; NHEJ, non-homologous end joining. *These two founders showed partial HDR, see Fig. 3.

**Table 3:** Modification of the rat *Foxp3* locus using Cas9 or TALEN proteins.

Form Cas9	ConcentrationCas9/sgRNA	N°. injectedeggs(% viable)	N°.transferredeggs	N° E15(%)^a^	N°.PCR GFP+(%)^a^ Tg	N°. HDR+ (%)^a^(%)^b^ HDR	N°. of indel+(%)^a^ (%)^b^NHEJ
Foxp3 Cas 9 protein*	3 μM/3 μM	215 (65.6)	120	29 (24.2)	1 (0.8)	1 (0.8) (3.5)	2 (1.6) (6.9)
Foxp3 Cas9 mRNA*	50 ng/μl 10 ng/μl	191(79.1)	151	52 (34.4)	1 (0.7)	0 (0)	4 (2.6) (7.7)

*Cas9 protein sgRNA preformed equimolar complexes or Cas9 mRNA mixed with sgRNA were microinjected at the indicated concentrations along with linearized donor DNA at 2 ng/μl. ^a^(% of transferred embryos). ^b^(% of E15 fetus). Tg, transgenics by random integration; HDR, homology-directed repair; NHEJ, non-homologous end joining; E15: embryos at day 15.

**Table 4:** Modification of the rat *Anks3* locus using Cas9 or TALEN proteins.

Form Cas9	Concentration	N°. injectedeggs(% viable)	N°.transferredeggs	N° pups(%)^a^	N°. HDR+ (%)^a^(%)^b^ HDR	N°. of indel+pups (%)^a^ (%)^b^NHEJ
Ansk3 Cas9 protein *	3 μM/1.5 μM	227 (69.6)	156	22 (14.1)	4# (2.6) (18.2)	4 (2.6) (18.2)
Anks3 Cas9 mRNA*	50 ng/μl 5 ng/μl	154 (70.1)	105	17 (16.2)	1## (0.95) (5.9)	7 (6.7) (41.2)

*Cas9 protein sgRNA preformed equimolar complexes or Cas9 mRNA mixed with sgRNA were microinjected at the indicated concentrations along with linearized donor ssODNs at 7.5 ng/μl. ^a^(% of transferred embryos). ^b^(% of pups). #2 animals with faithful HDR+ in both alleles and the other 2 with HDR in one allele; ##partial insertion of donor DNA in one allele. HDR, homology-directed repair; NHEJ, non-homologous end joining.
