# Supplementary material for: Homology-directed repair in rodent zygotes using Cas9 and TALEN engineered proteins
Source: Sci Rep. 2015 Oct 7;5:14410. doi: 10.1038/srep14410 (PMC4595769; doi:10.1038/srep14410)
Supplement: Supplementary Information 1 [file srep14410-s1.doc]

**Homology-directed repair in rodent zygotes using Cas9 and TALEN engineered proteins.**

Séverine Ménoret^1,2 #^, Anne De Cian^3#^, Laurent Tesson^1,2^, Séverine Remy^1,2^, Claire Usal^1,2^, Jean-Baptiste Boulé^3^, Charlotte Boix^3^, Sandra Fontanière^4^, Alison Crénéguy^1^, Tuan H. Nguyen^1^, Lucas Brusselle ^1^, Reynald Thinard^1,2^, Dominique Gauguier^5,6^, Jean-Paul Concordet^3^, Yacine Cherifi^4^, Alexandre Fraichard^4^, Carine Giovannangeli^3^, Ignacio Anegon^1,2*^.

**SUPPLEMENTARY INFORMATION**

**Table S1. Primer sequences.**

**Rat *Rosa26***

| **Primer name** | **5’-3’ Sequence** |
| --- | --- |
| ***NHEJ detection***  rROSAfw1  rROSArev1 | TGAACTGTGAATAGGCCCAAGTG  GCATTTTAAAAGAGCCCAGTACTTCA |
| ***Donor insertion***  GFP Up  GFP Lo3 | CCTCGTGACCACCCTGACCT  TCCATGCCGAGAGTGATCCC |
| ***In/out PCR***  rROSA26-5outFor  rROSA26-3outRev  5CAGpRev  3BGHpA-Up2 | TCCCACCCTCCCCTTCCTCT  TGGGTATCACTGGCTGTCCTAGATA  GGCTATGAACTAATGACCCCGTAAT  CCAGATTTTTCCTCCTCTCCTG |

**Mouse *Rosa26*.**

| **Primer name** | **5’-3’ Sequence** |
| --- | --- |
| **NHEJ detection**   | **mROSAfw1**  **mROSArev1** |  | | --- | --- | | **CAATACCTTTCTGGGAGTTCTCTGC**  **CTGCATAAAACCCCAGATGACTACC** |
| **Donor insertion**  **Up**  **Low** | **CAATACCTTTCTGGGAGTTCTCTGC**  **GGTAGTCATCTGGGGTTTTATGCAG**  **GGACTCATGGTGGTACTCTTCTCACTGC** |
| **In/out PCR**  **Up**  **Low** | **AAGACGAAAAGGGCAAGCATCTTCC**  **GGACTCATGGTGGTACTCTTCTCACTGC**  **GGCGTTACTATGGGAACATACGTCATTATTG**  **GCTGGTCTAAATGTGATTTTGCCAAGC** |

**Rat *Foxp3***.

| **Primer name** | **5’-3’ Sequence** |
| --- | --- |
| ***NHEJ detection***  rFoxp3Up  rFoxp3Lo | aacctggggctaaatgtgtg  tagggtttgggttgagtcca |
| ***Donor insertion***  GFP Up  GFP Lo3 | CCTCGTGACCACCCTGACCT  TCCATGCCGAGAGTGATCCC |
| ***In/out PCR***  rFoxP3Ex8Up  rFoxP3HA3outRev | TCGTAGCCACCAGCACCCA  TGAACCCCCTTATGCCTCCCAG |

**Rat *Anks3***.

| **Primer name** | **5’-3’ Sequence** |
| --- | --- |
| ***NHEJ detection***  rAnks3Up  rAnks3Lo | ggcagaaatggaaactggaa  caaaggtccagcatacagca |

**Sequences**

Sequences obtained for rat *Rosa26* TALEN subunit expression in *E.coli* are the following (ORF in capital letters):

>pSKB3-ROSA-L

gatctcgatcccgcgaaattaatacgactcactataggggaattgtgagcggataacaattcccctctagaaataatttt

gtttaactttaagaaggagatataccATGGGCAGCAGCCATCATCATCATCATCACGATTACGATATCCCAACGACCGAA

AACCTTTACTTCCAGGGCCATATGGCTCCAAAGAAGAAGCGTAAGGTATACCCATACGATGTTCCTGACTATGCGGGCTA

TCCCTATGACGTCCCGGACTATGCAGGATCGTATCCATATGACGTTCCAGATTACGCTGCTCATGGTACCGTGGATCTAC

GCACGCTCGGCTACAGCCAGCAGCAACAGGAGAAGATCAAACCGAAGGTTCGTTCGACAGTGGCGCAGCACCACGAGGCA

CTGGTCGGCCATGGGTTTACACACGCGCACATCGTTGCGCTCAGCCAACACCCGGCAGCGTTAGGGACCGTCGCTGTCAA

GTATCAGGACATGATCGCAGCGTTGCCAGAGGCGACACACGAAGCGATCGTTGGCGTCGGCAAACAGTGGTCCGGCGCAC

GCGCTCTGGAGGCCTTGCTCACGGTGGCGGGAGAGTTGAGAGGTCCACCGTTACAGTTGGACACAGGCCAACTTCTCAAG

ATTGCAAAACGTGGCGGCGTGACCGCAGTGGAGGCAGTGCATGCATGGCGCAATGCACTGACGGGTGCCCCCCTGAACCT

GACCCCGGAGCAGGTGGTGGCCATCGCTAGTAATATTGGTGGCAAACAGGCTCTTGAGACGGTTCAGCGCCTCCTTCCAG

TTCTCTGTCAAGCCCACGGACTCACCCCAGATCAAGTTGTAGCGATTGCTAGTCATGACGGTGGCAAACAGGCTCTTGAG

ACCGTCCAACGCCTTCTACCAGTTCTCTGTCAAGCCCACGGACTAACCCCAGCGCAAGTTGTAGCGATTGCTAGTAATAT

TGGTGGCAAACAGGCTCTTGAGACGGTTCAGCGCCTCCTTCCAGTTCTCTGTCAAGCCCACGGACTCACCCCAGATCAAG

TTGTAGCGATTGCTAGTAACAATGGTGGCAAACAGGCTCTTGAAACCGTACAGCGCCTACTGCCAGTTCTCTGTCAAGCC

CACGGTCTGACTCCGGAGCAAGTTGTAGCGATTGCTAGTCATGACGGTGGCAAACAGGCTCTTGAGACCGTCCAACGCCT

TCTACCAGTTCTCTGTCAAGCCCACGGACTAACCCCAGCGCAAGTTGTAGCGATTGCTAGTCATGACGGTGGCAAACAGG

CTCTTGAGACCGTCCAACGCCTTCTACCAGTTCTCTGTCAAGCCCACGGACTAACCCCAGCGCAAGTTGTAGCGATTGCT

AGTAATGGGGGTGGCAAACAGGCTCTTGAGACTGTTCAGCGCCTTCTACCAGTTCTCTGTCAAGCCCACGGCCTGACGCC

CGAGCAAGTTGTAGCGATTGCTAGTCATGACGGTGGCAAACAGGCTCTTGAGACCGTCCAACGCCTTCTACCAGTTCTCT

GTCAAGCCCACGGACTAACCCCAGCGCAAGTTGTAGCGATTGCTAGTAACAATGGTGGCAAACAGGCTCTTGAAACCGTA

CAGCGCCTACTGCCAGTTCTCTGTCAAGCCCACGGTCTGACTCCGGAGCAAGTTGTAGCGATTGCTAGTAATATTGGTGG

CAAACAGGCTCTTGAGACGGTTCAGCGCCTCCTTCCAGTTCTCTGTCAAGCCCACGGACTCACCCCAGATCAAGTTGTAG

CGATTGCTAGTAATGGGGGTGGCAAACAGGCTCTTGAGACTGTTCAGCGCCTTCTACCAGTTCTCTGTCAAGCCCACGGC

CTGACGCCCGAGCAAGTTGTAGCGATTGCTAGTAATGGGGGTGGCAAACAGGCTCTTGAGACTGTTCAGCGCCTTCTACC

AGTTCTCTGTCAAGCCCACGGCCTGACGCCCGAGCAAGTTGTAGCGATTGCTAGTAATGGGGGTGGCAAACAGGCTCTTG

AGACTGTTCAGCGCCTTCTACCAGTTCTCTGTCAAGCCCACGGCCTGACGCCCGAGCAAGTTGTAGCGATTGCTAGTAAC

AATGGTGGCAAACAGGCTCTTGAAACCGTACAGCGCCTACTGCCAGTTCTCTGTCAAGCCCACGGTCTGACTCCGGAGCA

AGTTGTAGCGATTGCTAGTAATGGGGGTGGCAAACAGGCTCTTGAGACTGTTCAGCGCCTTCTACCAGTTCTCTGTCAAG

CCCACGGCCTGACGCCCGAGCAAGTTGTAGCGATTGCTAGTAACAATGGTGGCAAACAGGCTCTTGAAACCGTACAGCGC

CTACTGCCAGTTCTCTGTCAAGCCCACGGTCTGACTCCGGAGCAAGTTGTAGCGATTGCTAGCAACAATGGCGGCAGGCC

GGCGCTGGAGAGCATTGTTGCCCAGTTATCTCGCCCTGATCCGGCGTTGGCCGCGTTGACCAACGACCACCTCGTCGCCT

TGGCCTGCCTCGGCGGACGTCCTGCGCTGGATGCAGTGAAAAAGGGATTGCCGCACGCGCCGGCCTTGATCAAAAGAACC

AATCGCCGTATTCCCGAACGCACATCCCATCGCGTTGCCGGATCCCAGCTGGTGAAGAGCGAGCTGGAGGAGAAGAAGAG

CGAGCTGAGACACAAGCTGAAGTACGTGCCCCACGAGTACATCGAGCTGATCGAGATCGCCAGAAACAGCACCCAGGACA

GAATCCTGGAGATGAAGGTGATGGAGTTCTTCATGAAGGTGTACGGCTACAGAGGCAAGCACCTGGGCGGCAGCAGAAAG

CCCGACGGCGCCATCTACACCGTGGGCAGCCCCATCGACTACGGCGTGATCGTGGACACCAAGGCCTACAGCGGCGGCTA

CAACCTGCCCATCGGCCAGGCCGACGAGATGCAGAGATACGTGGAGGAGAACCAGACCAGAAACAAGCACATCAACCCCA

ACGAGTGGTGGAAGGTGTACCCCAGCAGCGTGACCGAGTTCAAGTTCCTGTTCGTGAGCGGCCACTTCAAGGGCAACTAC

AAGGCCCAGCTGACCAGACTGAACCACATCACCAACTGCAACGGCGCCGTGCTGAGCGTGGAGGAGCTGCTGATCGGCGG

CGAGATGATCAAGGCCGGCACCCTGACCCTGGAGGAGGTGAGAAGAAAGTTCAACAACGGCGAGATCAACTTCAGAAGCT

GAtaactggagctctaactagcataaccccttggggcctctaaacgggtcttgaggggttttttgctgaaaggaggaact

atatccggattggcgaatgggacgcgccctgtagcggcgcattaagcgcggcgggtgtggtggttacgcgcagcgtgacc

gctacacttgccagcgccctagcgcccgctcctttcgctttcttcccttcctttctcgccacgttcgccggctttccccg

tcaagctctaaatcgggggctccctttagggttccgatttagtgctttacggcacctcgaccccaaaaaacttgattagg

gtgatggttcacgtagtgggccatcgccctgatagacggtttttcgccctttgacgttggagtccacgttctttaatagt

ggactcttgttccaaactggaacaacactcaaccctatctcggtctattcttttgatttataagggattttgccgatttc

ggcctattggttaaaaaatgagctgatttaacaaaaatttaacgcgaattttaacaaaatattaacgtttacaatttcag

gtggcacttttcggggaaatgtgcgcggaacccctatttgtttatttttctaaatacattcaaatatgtatccgctcatg

aattaattcttagaaaaactcatcgagcatcaaatgaaactgcaatttattcatatcaggattatcaataccatattttt

gaaaaagccgtttctgtaatgaaggagaaaactcaccgaggcagttccataggatggcaagatcctggtatcggtctgcg

attccgactcgtccaacatcaatacaacctattaatttcccctcgtcaaaaataaggttatcaagtgagaaatcaccatg

agtgacgactgaatccggtgagaatggcaaaagtttatgcatttctttccagacttgttcaacaggccagccattacgct

cgtcatcaaaatcactcgcatcaaccaaaccgttattcattcgtgattgcgcctgagcgagacgaaatacgcgatcgctg

ttaaaaggacaattacaaacaggaatcgaatgcaaccggcgcaggaacactgccagcgcatcaacaatattttcacctga

atcaggatattcttctaatacctggaatgctgttttcccggggatcgcagtggtgagtaaccatgcatcatcaggagtac

ggataaaatgcttgatggtcggaagaggcataaattccgtcagccagtttagtctgaccatctcatctgtaacatcattg

gcaacgctacctttgccatgtttcagaaacaactctggcgcatcgggcttcccatacaatcgatagattgtcgcacctga

ttgcccgacattatcgcgagcccatttatacccatataaatcagcatccatgttggaatttaatcgcggcctagagcaag

acgtttcccgttgaatatggctcataacaccccttgtattactgtttatgtaagcagacagttttattgttcatgaccaa

aatcccttaacgtgagttttcgttccactgagcgtcagaccccgtagaaaagatcaaaggatcttcttgagatccttttt

ttctgcgcgtaatctgctgcttgcaaacaaaaaaaccaccgctaccagcggtggtttgtttgccggatcaagagctacca

actctttttccgaaggtaactggcttcagcagagcgcagataccaaatactgtccttctagtgtagccgtagttaggcca

ccacttcaagaactctgtagcaccgcctacatacctcgctctgctaatcctgttaccagtggctgctgccagtggcgata

agtcgtgtcttaccgggttggactcaagacgatagttaccggataaggcgcagcggtcgggctgaacggggggttcgtgc

acacagcccagcttggagcgaacgacctacaccgaactgagatacctacagcgtgagctatgagaaagcgccacgcttcc

cgaagggagaaaggcggacaggtatccggtaagcggcagggtcggaacaggagagcgcacgagggagcttccagggggaa

acgcctggtatctttatagtcctgtcgggtttcgccacctctgacttgagcgtcgatttttgtgatgctcgtcagggggg

cggagcctatggaaaaacgccagcaacgcggcctttttacggttcctggccttttgctggccttttgctcacatgttctt

tcctgcgttatcccctgattctgtggataaccgtattaccgcctttgagtgagctgataccgctcgccgcagccgaacga

ccgagcgcagcgagtcagtgagcgaggaagcggaagagcgcctgatgcggtattttctccttacgcatctgtgcggtatt

tcacaccgcatatatggtgcactctcagtacaatctgctctgatgccgcatagttaagccagtatacactccgctatcgc

tacgtgactgggtcatggctgcgccccgacacccgccaacacccgctgacgcgccctgacgggcttgtctgctcccggca

tccgcttacagacaagctgtgaccgtctccgggagctgcatgtgtcagaggttttcaccgtcatcaccgaaacgcgcgag

gcagctgcggtaaagctcatcagcgtggtcgtgaagcgattcacagatgtctgcctgttcatccgcgtccagctcgttga

gtttctccagaagcgttaatgtctggcttctgataaagcgggccatgttaagggcggttttttcctgtttggtcactgat

gcctccgtgtaagggggatttctgttcatgggggtaatgataccgatgaaacgagagaggatgctcacgatacgggttac

tgatgatgaacatgcccggttactggaacgttgtgagggtaaacaactggcggtatggatgcggcgggaccagagaaaaa

tcactcagggtcaatgccagcgcttcgttaatacagatgtaggtgttccacagggtagccagcagcatcctgcgatgcag

atccggaacataatggtgcagggcgctgacttccgcgtttccagactttacgaaacacggaaaccgaagaccattcatgt

tgttgctcaggtcgcagacgttttgcagcagcagtcgcttcacgttcgctcgcgtatcggtgattcattctgctaaccag

taaggcaaccccgccagcctagccgggtcctcaacgacaggagcacgatcatgcgcacccgtggggccgccatgccggcg

ataatggcctgcttctcgccgaaacgtttggtggcgggaccagtgacgaaggcttgagcgagggcgtgcaagattccgaa

taccgcaagcgacaggccgatcatcgtcgcgctccagcgaaagcggtcctcgccgaaaatgacccagagcgctgccggca

cctgtcctacgagttgcatgataaagaagacagtcataagtgcggcgacgatagtcatgccccgcgcccaccggaaggag

ctgactgggttgaaggctctcaagggcatcggtcgagatcccggtgcctaatgagtgagctaacttacattaattgcgtt

gcgctcactgcccgctttccagtcgggaaacctgtcgtgccagctgcattaatgaatcggccaacgcgcggggagaggcg

gtttgcgtattgggcgccagggtggtttttcttttcaccagtgagacgggcaacagctgattgcccttcaccgcctggcc

ctgagagagttgcagcaagcggtccacgctggtttgccccagcaggcgaaaatcctgtttgatggtggttaacggcggga

tataacatgagctgtcttcggtatcgtcgtatcccactaccgagatatccgcaccaacgcgcagcccggactcggtaatg

gcgcgcattgcgcccagcgccatctgatcgttggcaaccagcatcgcagtgggaacgatgccctcattcagcatttgcat

ggtttgttgaaaaccggacatggcactccagtcgccttcccgttccgctatcggctgaatttgattgcgagtgagatatt

tatgccagccagccagacgcagacgcgccgagacagaacttaatgggcccgctaacagcgcgatttgctggtgacccaat

gcgaccagatgctccacgcccagtcgcgtaccgtcttcatgggagaaaataatactgttgatgggtgtctggtcagagac

atcaagaaataacgccggaacattagtgcaggcagcttccacagcaatggcatcctggtcatccagcggatagttaatga

tcagcccactgacgcgttgcgcgagaagattgtgcaccgccgctttacaggcttcgacgccgcttcgttctaccatcgac

accaccacgctggcacccagttgatcggcgcgagatttaatcgccgcgacaatttgcgacggcgcgtgcagggccagact

ggaggtggcaacgccaatcagcaacgactgtttgcccgccagttgttgtgccacgcggttgggaatgtaattcagctccg

ccatcgccgcttccactttttcccgcgttttcgcagaaacgtggctggcctggttcaccacgcgggaaacggtctgataa

gagacaccggcatactctgcgacatcgtataacgttactggtttcacattcaccaccctgaattgactctcttccgggcg

ctatcatgccataccgcgaaaggttttgcgccattcgatggtgtccgggatctcgacgctctcccttatgcgactcctgc

attaggaagcagcccagtagtaggttgaggccgttgagcaccgccgccgcaaggaatggtgcatgcaaggagatggcgcc

caacagtcccccggccacggggcctgccaccatacccacgccgaaacaagcgctcatgagcccgaagtggcgagcccgat

cttccccatcggtgatgtcggcgatataggcgccagcaaccgcacctgtggcgccggtgatgccggccacgatgcgtccg

gcgtagaggatcga

>pSKB3-ROSA-R

gatctcgatcccgcgaaattaatacgactcactataggggaattgtgagcggataacaattcccctctagaaataatttt

gtttaactttaagaaggagatataccATGGGCAGCAGCCATCATCATCATCATCACGATTACGATATCCCAACGACCGAA

AACCTTTACTTCCAGGGCCATATGGCTCCAAAGAAGAAGCGTAAGGTATACCCATACGATGTTCCTGACTATGCGGGCTA

TCCCTATGACGTCCCGGACTATGCAGGATCGTATCCATATGACGTTCCAGATTACGCTGCTCATGGTACCGTGGATCTAC

GCACGCTCGGCTACAGCCAGCAGCAACAGGAGAAGATCAAACCGAAGGTTCGTTCGACAGTGGCGCAGCACCACGAGGCA

CTGGTCGGCCATGGGTTTACACACGCGCACATCGTTGCGCTCAGCCAACACCCGGCAGCGTTAGGGACCGTCGCTGTCAA

GTATCAGGACATGATCGCAGCGTTGCCAGAGGCGACACACGAAGCGATCGTTGGCGTCGGCAAACAGTGGTCCGGCGCAC

GCGCTCTGGAGGCCTTGCTCACGGTGGCGGGAGAGTTGAGAGGTCCACCGTTACAGTTGGACACAGGCCAACTTCTCAAG

ATTGCAAAACGTGGCGGCGTGACCGCAGTGGAGGCAGTGCATGCATGGCGCAATGCACTGACGGGTGCCCCCCTGAACCT

GACCCCGGAGCAGGTGGTGGCCATCGCTAGTAACAATGGTGGCAAACAGGCTCTTGAAACCGTACAGCGCCTACTGCCAG

TTCTCTGTCAAGCCCACGGTCTGACTCCGGAGCAAGTTGTAGCGATTGCTAGTAATATTGGTGGCAAACAGGCTCTTGAG

ACGGTTCAGCGCCTCCTTCCAGTTCTCTGTCAAGCCCACGGACTCACCCCAGATCAAGTTGTAGCGATTGCTAGTCATGA

CGGTGGCAAACAGGCTCTTGAGACCGTCCAACGCCTTCTACCAGTTCTCTGTCAAGCCCACGGACTAACCCCAGCGCAAG

TTGTAGCGATTGCTAGTAATGGGGGTGGCAAACAGGCTCTTGAGACTGTTCAGCGCCTTCTACCAGTTCTCTGTCAAGCC

CACGGCCTGACGCCCGAGCAAGTTGTAGCGATTGCTAGTCATGACGGTGGCAAACAGGCTCTTGAGACCGTCCAACGCCT

TCTACCAGTTCTCTGTCAAGCCCACGGACTAACCCCAGCGCAAGTTGTAGCGATTGCTAGTAATATTGGTGGCAAACAGG

CTCTTGAGACGGTTCAGCGCCTCCTTCCAGTTCTCTGTCAAGCCCACGGACTCACCCCAGATCAAGTTGTAGCGATTGCT

AGTAATGGGGGTGGCAAACAGGCTCTTGAGACTGTTCAGCGCCTTCTACCAGTTCTCTGTCAAGCCCACGGCCTGACGCC

CGAGCAAGTTGTAGCGATTGCTAGTAACAATGGTGGCAAACAGGCTCTTGAAACCGTACAGCGCCTACTGCCAGTTCTCT

GTCAAGCCCACGGTCTGACTCCGGAGCAAGTTGTAGCGATTGCTAGTAATATTGGTGGCAAACAGGCTCTTGAGACGGTT

CAGCGCCTCCTTCCAGTTCTCTGTCAAGCCCACGGACTCACCCCAGATCAAGTTGTAGCGATTGCTAGTAATATTGGTGG

CAAACAGGCTCTTGAGACGGTTCAGCGCCTCCTTCCAGTTCTCTGTCAAGCCCACGGACTCACCCCAGATCAAGTTGTAG

CGATTGCTAGTAATATTGGTGGCAAACAGGCTCTTGAGACGGTTCAGCGCCTCCTTCCAGTTCTCTGTCAAGCCCACGGA

CTCACCCCAGATCAAGTTGTAGCGATTGCTAGTCATGACGGTGGCAAACAGGCTCTTGAGACCGTCCAACGCCTTCTACC

AGTTCTCTGTCAAGCCCACGGACTAACCCCAGCGCAAGTTGTAGCGATTGCTAGTCATGACGGTGGCAAACAGGCTCTTG

AGACCGTCCAACGCCTTCTACCAGTTCTCTGTCAAGCCCACGGACTAACCCCAGCGCAAGTTGTAGCGATTGCTAGTAAT

ATTGGTGGCAAACAGGCTCTTGAGACGGTTCAGCGCCTCCTTCCAGTTCTCTGTCAAGCCCACGGACTCACCCCAGATCA

AGTTGTAGCGATTGCTAGTAACAATGGTGGCAAACAGGCTCTTGAAACCGTACAGCGCCTACTGCCAGTTCTCTGTCAAG

CCCACGGTCTGACTCCGGAGCAAGTTGTAGCGATTGCTAGTAATATTGGTGGCAAACAGGCTCTTGAGACGGTTCAGCGC

CTCCTTCCAGTTCTCTGTCAAGCCCACGGACTCACCCCAGATCAAGTTGTAGCGATTGCTAGCCATGACGGCGGCAGGCC

GGCGCTGGAGAGCATTGTTGCCCAGTTATCTCGCCCTGATCCGGCGTTGGCCGCGTTGACCAACGACCACCTCGTCGCCT

TGGCCTGCCTCGGCGGACGTCCTGCGCTGGATGCAGTGAAAAAGGGATTGCCGCACGCGCCGGCCTTGATCAAAAGAACC

AATCGCCGTATTCCCGAACGCACATCCCATCGCGTTGCCGGATCCCAGCTGGTGAAGAGCGAGCTGGAGGAGAAGAAGAG

CGAGCTGAGACACAAGCTGAAGTACGTGCCCCACGAGTACATCGAGCTGATCGAGATCGCCAGAAACAGCACCCAGGACA

GAATCCTGGAGATGAAGGTGATGGAGTTCTTCATGAAGGTGTACGGCTACAGAGGCAAGCACCTGGGCGGCAGCAGAAAG

CCCGACGGCGCCATCTACACCGTGGGCAGCCCCATCGACTACGGCGTGATCGTGGACACCAAGGCCTACAGCGGCGGCTA

CAACCTGCCCATCGGCCAGGCCGACGAGATGCAGAGATACGTGGAGGAGAACCAGACCAGAAACAAGCACATCAACCCCA

ACGAGTGGTGGAAGGTGTACCCCAGCAGCGTGACCGAGTTCAAGTTCCTGTTCGTGAGCGGCCACTTCAAGGGCAACTAC

AAGGCCCAGCTGACCAGACTGAACCACATCACCAACTGCAACGGCGCCGTGCTGAGCGTGGAGGAGCTGCTGATCGGCGG

CGAGATGATCAAGGCCGGCACCCTGACCCTGGAGGAGGTGAGAAGAAAGTTCAACAACGGCGAGATCAACTTCAGAAGCT

GAtaactggagctctaactagcataaccccttggggcctctaaacgggtcttgaggggttttttgctgaaaggaggaact

atatccggattggcgaatgggacgcgccctgtagcggcgcattaagcgcggcgggtgtggtggttacgcgcagcgtgacc

gctacacttgccagcgccctagcgcccgctcctttcgctttcttcccttcctttctcgccacgttcgccggctttccccg

tcaagctctaaatcgggggctccctttagggttccgatttagtgctttacggcacctcgaccccaaaaaacttgattagg

gtgatggttcacgtagtgggccatcgccctgatagacggtttttcgccctttgacgttggagtccacgttctttaatagt

ggactcttgttccaaactggaacaacactcaaccctatctcggtctattcttttgatttataagggattttgccgatttc

ggcctattggttaaaaaatgagctgatttaacaaaaatttaacgcgaattttaacaaaatattaacgtttacaatttcag

gtggcacttttcggggaaatgtgcgcggaacccctatttgtttatttttctaaatacattcaaatatgtatccgctcatg

aattaattcttagaaaaactcatcgagcatcaaatgaaactgcaatttattcatatcaggattatcaataccatattttt

gaaaaagccgtttctgtaatgaaggagaaaactcaccgaggcagttccataggatggcaagatcctggtatcggtctgcg

attccgactcgtccaacatcaatacaacctattaatttcccctcgtcaaaaataaggttatcaagtgagaaatcaccatg

agtgacgactgaatccggtgagaatggcaaaagtttatgcatttctttccagacttgttcaacaggccagccattacgct

cgtcatcaaaatcactcgcatcaaccaaaccgttattcattcgtgattgcgcctgagcgagacgaaatacgcgatcgctg

ttaaaaggacaattacaaacaggaatcgaatgcaaccggcgcaggaacactgccagcgcatcaacaatattttcacctga

atcaggatattcttctaatacctggaatgctgttttcccggggatcgcagtggtgagtaaccatgcatcatcaggagtac

ggataaaatgcttgatggtcggaagaggcataaattccgtcagccagtttagtctgaccatctcatctgtaacatcattg

gcaacgctacctttgccatgtttcagaaacaactctggcgcatcgggcttcccatacaatcgatagattgtcgcacctga

ttgcccgacattatcgcgagcccatttatacccatataaatcagcatccatgttggaatttaatcgcggcctagagcaag

acgtttcccgttgaatatggctcataacaccccttgtattactgtttatgtaagcagacagttttattgttcatgaccaa

aatcccttaacgtgagttttcgttccactgagcgtcagaccccgtagaaaagatcaaaggatcttcttgagatccttttt

ttctgcgcgtaatctgctgcttgcaaacaaaaaaaccaccgctaccagcggtggtttgtttgccggatcaagagctacca

actctttttccgaaggtaactggcttcagcagagcgcagataccaaatactgtccttctagtgtagccgtagttaggcca

ccacttcaagaactctgtagcaccgcctacatacctcgctctgctaatcctgttaccagtggctgctgccagtggcgata

agtcgtgtcttaccgggttggactcaagacgatagttaccggataaggcgcagcggtcgggctgaacggggggttcgtgc

acacagcccagcttggagcgaacgacctacaccgaactgagatacctacagcgtgagctatgagaaagcgccacgcttcc

cgaagggagaaaggcggacaggtatccggtaagcggcagggtcggaacaggagagcgcacgagggagcttccagggggaa

acgcctggtatctttatagtcctgtcgggtttcgccacctctgacttgagcgtcgatttttgtgatgctcgtcagggggg

cggagcctatggaaaaacgccagcaacgcggcctttttacggttcctggccttttgctggccttttgctcacatgttctt

tcctgcgttatcccctgattctgtggataaccgtattaccgcctttgagtgagctgataccgctcgccgcagccgaacga

ccgagcgcagcgagtcagtgagcgaggaagcggaagagcgcctgatgcggtattttctccttacgcatctgtgcggtatt

tcacaccgcatatatggtgcactctcagtacaatctgctctgatgccgcatagttaagccagtatacactccgctatcgc

tacgtgactgggtcatggctgcgccccgacacccgccaacacccgctgacgcgccctgacgggcttgtctgctcccggca

tccgcttacagacaagctgtgaccgtctccgggagctgcatgtgtcagaggttttcaccgtcatcaccgaaacgcgcgag

gcagctgcggtaaagctcatcagcgtggtcgtgaagcgattcacagatgtctgcctgttcatccgcgtccagctcgttga

gtttctccagaagcgttaatgtctggcttctgataaagcgggccatgttaagggcggttttttcctgtttggtcactgat

gcctccgtgtaagggggatttctgttcatgggggtaatgataccgatgaaacgagagaggatgctcacgatacgggttac

tgatgatgaacatgcccggttactggaacgttgtgagggtaaacaactggcggtatggatgcggcgggaccagagaaaaa

tcactcagggtcaatgccagcgcttcgttaatacagatgtaggtgttccacagggtagccagcagcatcctgcgatgcag

atccggaacataatggtgcagggcgctgacttccgcgtttccagactttacgaaacacggaaaccgaagaccattcatgt

tgttgctcaggtcgcagacgttttgcagcagcagtcgcttcacgttcgctcgcgtatcggtgattcattctgctaaccag

taaggcaaccccgccagcctagccgggtcctcaacgacaggagcacgatcatgcgcacccgtggggccgccatgccggcg

ataatggcctgcttctcgccgaaacgtttggtggcgggaccagtgacgaaggcttgagcgagggcgtgcaagattccgaa

taccgcaagcgacaggccgatcatcgtcgcgctccagcgaaagcggtcctcgccgaaaatgacccagagcgctgccggca

cctgtcctacgagttgcatgataaagaagacagtcataagtgcggcgacgatagtcatgccccgcgcccaccggaaggag

ctgactgggttgaaggctctcaagggcatcggtcgagatcccggtgcctaatgagtgagctaacttacattaattgcgtt

gcgctcactgcccgctttccagtcgggaaacctgtcgtgccagctgcattaatgaatcggccaacgcgcggggagaggcg

gtttgcgtattgggcgccagggtggtttttcttttcaccagtgagacgggcaacagctgattgcccttcaccgcctggcc

ctgagagagttgcagcaagcggtccacgctggtttgccccagcaggcgaaaatcctgtttgatggtggttaacggcggga

tataacatgagctgtcttcggtatcgtcgtatcccactaccgagatatccgcaccaacgcgcagcccggactcggtaatg

gcgcgcattgcgcccagcgccatctgatcgttggcaaccagcatcgcagtgggaacgatgccctcattcagcatttgcat

ggtttgttgaaaaccggacatggcactccagtcgccttcccgttccgctatcggctgaatttgattgcgagtgagatatt

tatgccagccagccagacgcagacgcgccgagacagaacttaatgggcccgctaacagcgcgatttgctggtgacccaat

gcgaccagatgctccacgcccagtcgcgtaccgtcttcatgggagaaaataatactgttgatgggtgtctggtcagagac

atcaagaaataacgccggaacattagtgcaggcagcttccacagcaatggcatcctggtcatccagcggatagttaatga

tcagcccactgacgcgttgcgcgagaagattgtgcaccgccgctttacaggcttcgacgccgcttcgttctaccatcgac

accaccacgctggcacccagttgatcggcgcgagatttaatcgccgcgacaatttgcgacggcgcgtgcagggccagact

ggaggtggcaacgccaatcagcaacgactgtttgcccgccagttgttgtgccacgcggttgggaatgtaattcagctccg

ccatcgccgcttccactttttcccgcgttttcgcagaaacgtggctggcctggttcaccacgcgggaaacggtctgataa

gagacaccggcatactctgcgacatcgtataacgttactggtttcacattcaccaccctgaattgactctcttccgggcg

ctatcatgccataccgcgaaaggttttgcgccattcgatggtgtccgggatctcgacgctctcccttatgcgactcctgc

attaggaagcagcccagtagtaggttgaggccgttgagcaccgccgccgcaaggaatggtgcatgcaaggagatggcgcc

caacagtcccccggccacggggcctgccaccatacccacgccgaaacaagcgctcatgagcccgaagtggcgagcccgat

cttccccatcggtgatgtcggcgatataggcgccagcaaccgcacctgtggcgccggtgatgccggccacgatgcgtccg

gcgtagaggatcga

Coding sequence for His-MBP –TEV-NLS-*S. pyogenes* Cas9-2NLS

>pMJ806-3NLS

ATGCACCATCACCATCACCATGGAAAAATCGAAGAAGGTAAACTGGTAATCTGGATTAACGGCGATAAAGGCTATAACGG

TCTCGCTGAAGTCGGTAAGAAATTCGAGAAAGATACCGGAATTAAAGTCACCGTTGAGCATCCGGATAAACTGGAAGAGA

AATTCCCACAGGTTGCGGCAACTGGCGATGGCCCTGACATTATCTTCTGGGCACACGACCGCTTTGGTGGCTACGCTCAA

TCTGGCCTGTTGGCTGAAATCACCCCGGACAAAGCGTTCCAGGACAAGCTGTATCCGTTTACCTGGGATGCCGTACGTTA

CAACGGCAAGCTGATTGCTTACCCGATCGCTGTTGAAGCGTTATCGCTGATTTATAACAAAGATCTGCTGCCGAACCCGC

CAAAAACCTGGGAAGAGATCCCGGCGCTGGATAAAGAACTGAAAGCGAAAGGTAAGAGCGCGCTGATGTTCAACCTGCAA

GAACCGTACTTCACCTGGCCGCTGATTGCTGCTGACGGGGGTTATGCGTTCAAGTATGAAAACGGCAAGTACGACATTAA

AGACGTGGGCGTGGATAACGCTGGCGCGAAAGCGGGTCTGACCTTCCTGGTTGACCTGATTAAAAACAAACACATGAATG

CAGACACCGATTACTCCATCGCAGAAGCTGCCTTTAATAAAGGCGAAACAGCGATGACCATCAACGGCCCGTGGGCATGG

TCCAACATCGACACCAGCAAAGTGAATTATGGTGTAACGGTACTGCCGACCTTCAAGGGTCAACCATCCAAACCGTTCGT

TGGCGTGCTGAGCGCAGGTATTAACGCCGCCAGTCCGAACAAAGAGCTGGCAAAAGAGTTCCTCGAAAACTATCTGCTGA

CTGATGAAGGTCTGGAAGCGGTTAATAAAGACAAACCGCTGGGTGCCGTAGCGCTGAAGTCTTACGAGGAAGAGTTGGCG

AAAGATCCACGTATTGCCGCCACTATGGAAAACGCCCAGAAAGGTGAAATCATGCCGAACATCCCGCAGATGTCCGCTTT

CTGGTATGCCGTGCGTACTGCGGTGATCAACGCCGCCAGCGGTCGTCAGACTGTCGATGAAGCCCTGAAAGACGCGCAGA

CTAATTCGAGCTCGAACAACAACAACACTAGTGAAAACCTGTATTTCCAGGGAGCAGCCTCGatggtgcctaagaagaag

agaaaggtgATGGATAAGAAATACTCAATAGGCTTAGATATCGGCACAAATAGCGTCGGATGGGCGGTGATCACTGATGA

ATATAAGGTTCCGTCTAAAAAGTTCAAGGTTCTGGGAAATACAGACCGCCACAGTATCAAAAAAAATCTTATAGGGGCTC

TTTTATTTGACAGTGGAGAGACAGCGGAAGCGACTCGTCTCAAACGGACAGCTCGTAGAAGGTATACACGTCGGAAGAAT

CGTATTTGTTATCTACAGGAGATTTTTTCAAATGAGATGGCGAAAGTAGATGATAGTTTCTTTCATCGACTTGAAGAGTC

TTTTTTGGTGGAAGAAGACAAGAAGCATGAACGTCATCCTATTTTTGGAAATATAGTAGATGAAGTTGCTTATCATGAGA

AATATCCAACTATCTATCATCTGCGAAAAAAATTGGTAGATTCTACTGATAAAGCGGATTTGCGCTTAATCTATTTGGCC

TTAGCGCATATGATTAAGTTTCGTGGTCATTTTTTGATTGAGGGAGATTTAAATCCTGATAATAGTGATGTGGACAAACT

ATTTATCCAGTTGGTACAAACCTACAATCAATTATTTGAAGAAAACCCTATTAACGCAAGTGGAGTAGATGCTAAAGCGA

TTCTTTCTGCACGATTGAGTAAATCAAGACGATTAGAAAATCTCATTGCTCAGCTCCCCGGTGAGAAGAAAAATGGCTTA

TTTGGGAATCTCATTGCTTTGTCATTGGGTTTGACCCCTAATTTTAAATCAAATTTTGATTTGGCAGAAGATGCTAAATT

ACAGCTTTCAAAAGATACTTACGATGATGATTTAGATAATTTATTGGCGCAAATTGGAGATCAATATGCTGATTTGTTTT

TGGCAGCTAAGAATTTATCAGATGCTATTTTACTTTCAGATATCCTAAGAGTAAATACTGAAATAACTAAGGCTCCCCTA

TCAGCTTCAATGATTAAACGCTACGATGAACATCATCAAGACTTGACTCTTTTAAAAGCTTTAGTTCGACAACAACTTCC

AGAAAAGTATAAAGAAATCTTTTTTGATCAATCAAAAAACGGATATGCAGGTTATATTGATGGGGGAGCTAGCCAAGAAG

AATTTTATAAATTTATCAAACCAATTTTAGAAAAAATGGATGGTACTGAGGAATTATTGGTGAAACTAAATCGTGAAGAT

TTGCTGCGCAAGCAACGGACCTTTGACAACGGCTCTATTCCCCATCAAATTCACTTGGGTGAGCTGCATGCTATTTTGAG

AAGACAAGAAGACTTTTATCCATTTTTAAAAGACAATCGTGAGAAGATTGAAAAAATCTTGACTTTTCGAATTCCTTATT

ATGTTGGTCCATTGGCGCGTGGCAATAGTCGTTTTGCATGGATGACTCGGAAGTCTGAAGAAACAATTACCCCATGGAAT

TTTGAAGAAGTTGTCGATAAAGGTGCTTCAGCTCAATCATTTATTGAACGCATGACAAACTTTGATAAAAATCTTCCAAA

TGAAAAAGTACTACCAAAACATAGTTTGCTTTATGAGTATTTTACGGTTTATAACGAATTGACAAAGGTCAAATATGTTA

CTGAAGGAATGCGAAAACCAGCATTTCTTTCAGGTGAACAGAAGAAAGCCATTGTTGATTTACTCTTCAAAACAAATCGA

AAAGTAACCGTTAAGCAATTAAAAGAAGATTATTTCAAAAAAATAGAATGTTTTGATAGTGTTGAAATTTCAGGAGTTGA

AGATAGATTTAATGCTTCATTAGGTACCTACCATGATTTGCTAAAAATTATTAAAGATAAAGATTTTTTGGATAATGAAG

AAAATGAAGATATCTTAGAGGATATTGTTTTAACATTGACCTTATTTGAAGATAGGGAGATGATTGAGGAAAGACTTAAA

ACATATGCTCACCTCTTTGATGATAAGGTGATGAAACAGCTTAAACGTCGCCGTTATACTGGTTGGGGACGTTTGTCTCG

AAAATTGATTAATGGTATTAGGGATAAGCAATCTGGCAAAACAATATTAGATTTTTTGAAATCAGATGGTTTTGCCAATC

GCAATTTTATGCAGCTGATCCATGATGATAGTTTGACATTTAAAGAAGACATTCAAAAAGCACAAGTGTCTGGACAAGGC

GATAGTTTACATGAACATATTGCAAATTTAGCTGGTAGCCCTGCTATTAAAAAAGGTATTTTACAGACTGTAAAAGTTGT

TGATGAATTGGTCAAAGTAATGGGGCGGCATAAGCCAGAAAATATCGTTATTGAAATGGCACGTGAAAATCAGACAACTC

AAAAGGGCCAGAAAAATTCGCGAGAGCGTATGAAACGAATCGAAGAAGGTATCAAAGAATTAGGAAGTCAGATTCTTAAA

GAGCATCCTGTTGAAAATACTCAATTGCAAAATGAAAAGCTCTATCTCTATTATCTCCAAAATGGAAGAGACATGTATGT

GGACCAAGAATTAGATATTAATCGTTTAAGTGATTATGATGTCGATCACATTGTTCCACAAAGTTTCCTTAAAGACGATT

CAATAGACAATAAGGTCTTAACGCGTTCTGATAAAAATCGTGGTAAATCGGATAACGTTCCAAGTGAAGAAGTAGTCAAA

AAGATGAAAAACTATTGGAGACAACTTCTAAACGCCAAGTTAATCACTCAACGTAAGTTTGATAATTTAACGAAAGCTGA

ACGTGGAGGTTTGAGTGAACTTGATAAAGCTGGTTTTATCAAACGCCAATTGGTTGAAACTCGCCAAATCACTAAGCATG

TGGCACAAATTTTGGATAGTCGCATGAATACTAAATACGATGAAAATGATAAACTTATTCGAGAGGTTAAAGTGATTACC

TTAAAATCTAAATTAGTTTCTGACTTCCGAAAAGATTTCCAATTCTATAAAGTACGTGAGATTAACAATTACCATCATGC

CCATGATGCGTATCTAAATGCCGTCGTTGGAACTGCTTTGATTAAGAAATATCCAAAACTTGAATCGGAGTTTGTCTATG

GTGATTATAAAGTTTATGATGTTCGTAAAATGATTGCTAAGTCTGAGCAAGAAATAGGCAAAGCAACCGCAAAATATTTC

TTTTACTCTAATATCATGAACTTCTTCAAAACAGAAATTACACTTGCAAATGGAGAGATTCGCAAACGCCCTCTAATCGA

AACTAATGGGGAAACTGGAGAAATTGTCTGGGATAAAGGGCGAGATTTTGCCACAGTGCGCAAAGTATTGTCCATGCCCC

AAGTCAATATTGTCAAGAAAACAGAAGTACAGACAGGCGGATTCTCCAAGGAGTCAATTTTACCAAAAAGAAATTCGGAC

AAGCTTATTGCTCGTAAAAAAGACTGGGATCCAAAAAAATATGGTGGTTTTGATAGTCCAACGGTAGCTTATTCAGTCCT

AGTGGTTGCTAAGGTGGAAAAAGGGAAATCGAAGAAGTTAAAATCCGTTAAAGAGTTACTAGGGATCACAATTATGGAAA

GAAGTTCCTTTGAAAAAAATCCGATTGACTTTTTAGAAGCTAAAGGATATAAGGAAGTTAAAAAAGACTTAATCATTAAA

CTACCTAAATATAGTCTTTTTGAGTTAGAAAACGGTCGTAAACGGATGCTGGCTAGTGCCGGAGAATTACAAAAAGGAAA

TGAGCTGGCTCTGCCAAGCAAATATGTGAATTTTTTATATTTAGCTAGTCATTATGAAAAGTTGAAGGGTAGTCCAGAAG

ATAACGAACAAAAACAATTGTTTGTGGAGCAGCATAAGCATTATTTAGATGAGATTATTGAGCAAATCAGTGAATTTTCT

AAGCGTGTTATTTTAGCAGATGCCAATTTAGATAAAGTTCTTAGTGCATATAACAAACATAGAGACAAACCAATACGTGA

ACAAGCAGAAAATATTATTCATTTATTTACGTTGACGAATCTTGGAGCTCCCGCTGCTTTTAAATATTTTGATACAACAA

TTGATCGTAAACGATATACGTCTACAAAAGAAGTTTTAGATGCCACTCTTATCCATCAATCCATCACTGGTCTTTATGAA

ACACGCATTGATTTGAGTCAGCTAGGAGGTGACGGTTCTCCCAAGAAGAAGAGGAAAGTCTCGAGCgcgaagaaaaagaa

gctggat
